# Supplementary material for: Turning main-group element magnesium into a highly active electrocatalyst for oxygen reduction reaction
Source: Nat Commun. 2020 Feb 18;11:938. doi: 10.1038/s41467-020-14565-w (PMC7028951; doi:10.1038/s41467-020-14565-w)
Supplement: Supplementary file 1 — Supplementary information [file 41467_2020_14565_MOESM1_ESM.pdf]

Turning main-group element Mg into a highly active electrocatalyst for oxygen reduction reaction

Liu et al.

Turning main-group element Mg into a highly active electrocatalyst for oxygen reduction reaction

Shuai Liu,[1] Zedong Li,[1] Changlai Wang, [1] Weiwei Tao,[3] Minxue Huang,[1] Ming Zuo,[1] Yang Yang,[1] Kang Yang,[1] Lijuan Zhang,[4] Shi Chen,[1] Pengping Xu,[1] Qianwang Chen[1,2]

[1]Hefei National Laboratory for Physical Science at Microscale and Department of Materials Science & Engineering, University of Science and Technology of China, Hefei 230026, China

[2]Anhui Province Key Laboratory of Condensed Matter Physics at Extreme Conditions, High Magnetic Field Laboratory of Chinese, Academy of Sciences, Hefei 230031, China

[3]Department of Mechanical Engineering, Boston University, Boston, MA 02215, USA

[4]Shanghai Synchrotron Radiation Facility, Shanghai Institute of Applied Physics, Shanghai 201203, China

Contents

1. Supplementary Figures 1-41

1.1 The calculated details for models (Supplementary Figures 1-11)

1.2 Characterization of pyrolysis products and ORR performance of the catalysts (Supplementary Figures 12-31)

1.3 Characterization of post-doped Mg in N-C host and ORR performance of the catalysts (Supplementary Figures 32-36)

1.4 Reaction pathway details and other spatial configurations similar to MN2C (Supplementary Figures 37-41)

2. Supplementary Tables 1-11

3. Supplementary References

1. Supplementary Figures

1.1 The calculated details for models

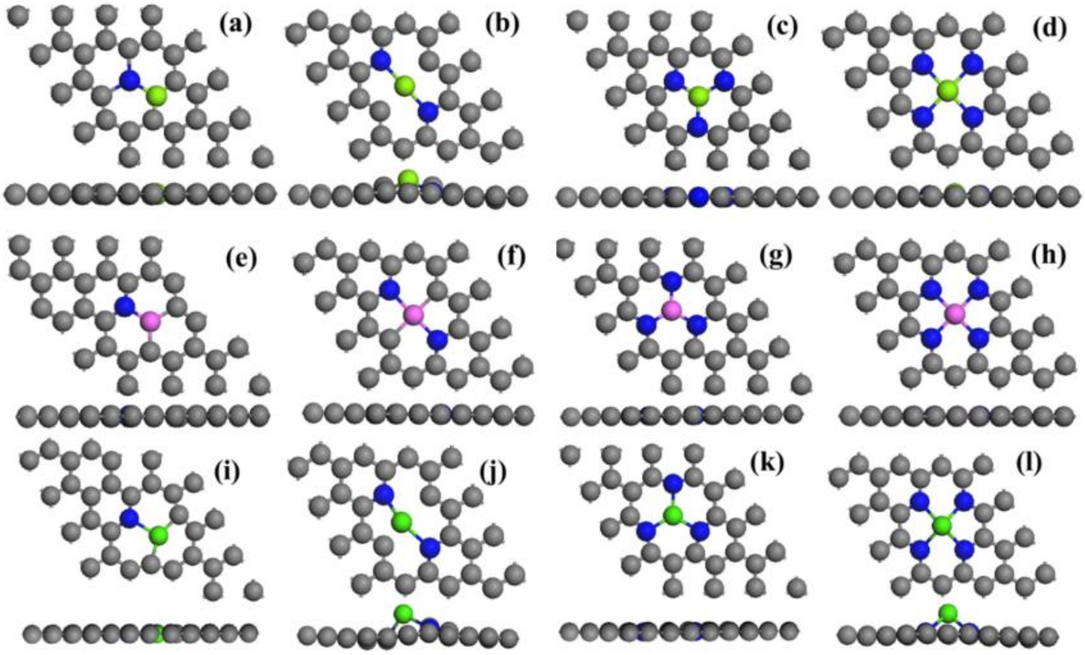

Supplementary Figure 1. Atomistic structure of the calculated models. (a-d) The MNxC model with one, two, three, four N atoms respectively from both the top view and side view. (e-f) The ANxC model with one, two, three, four N atoms respectively from both the top view and side view. (i-l) The CNxC model with one, two, three, four N atoms respectively from both the top view and side view. For all images, green, blue and gray balls represent metal, N and C atoms, respectively.

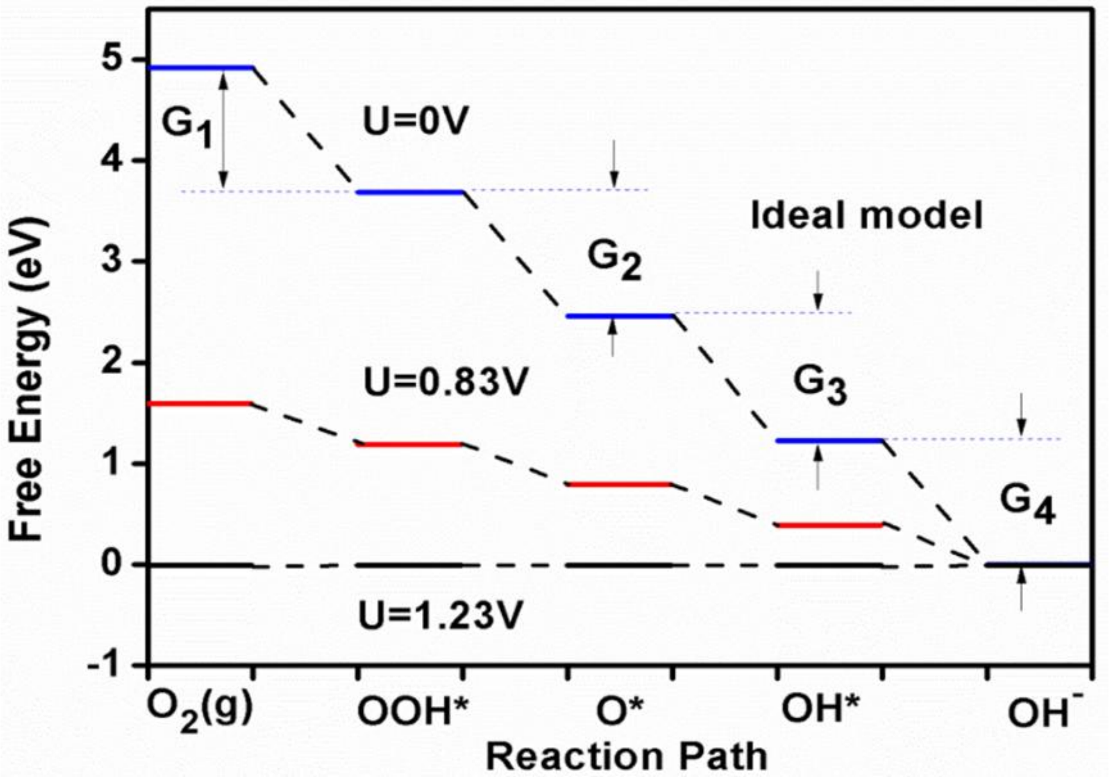

Supplementary Figure 2. Free-energy evolution diagram for ORR of the ideal model through a 4e- associative pathway under electrode potential of U=0, U=0.83V and U=1.23V.

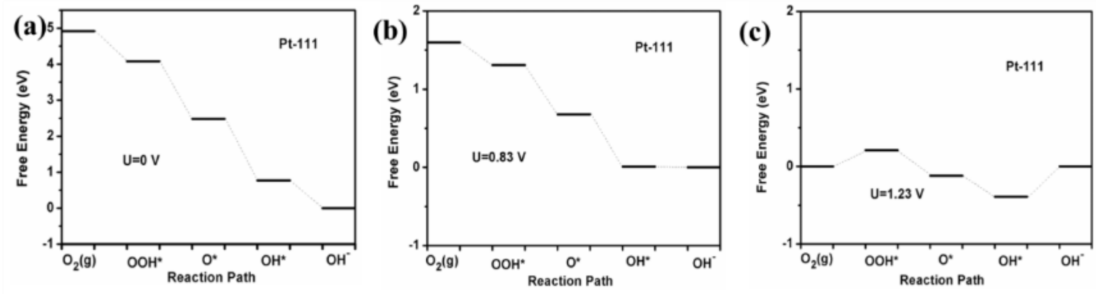

Supplementary Figure 3. Free-energy evolution diagram for ORR of Pt(111) model. It is shown with a 4e- associative pathway under output potential of (a) U=0, (b) U=0.8V and (c) U=1.23V.

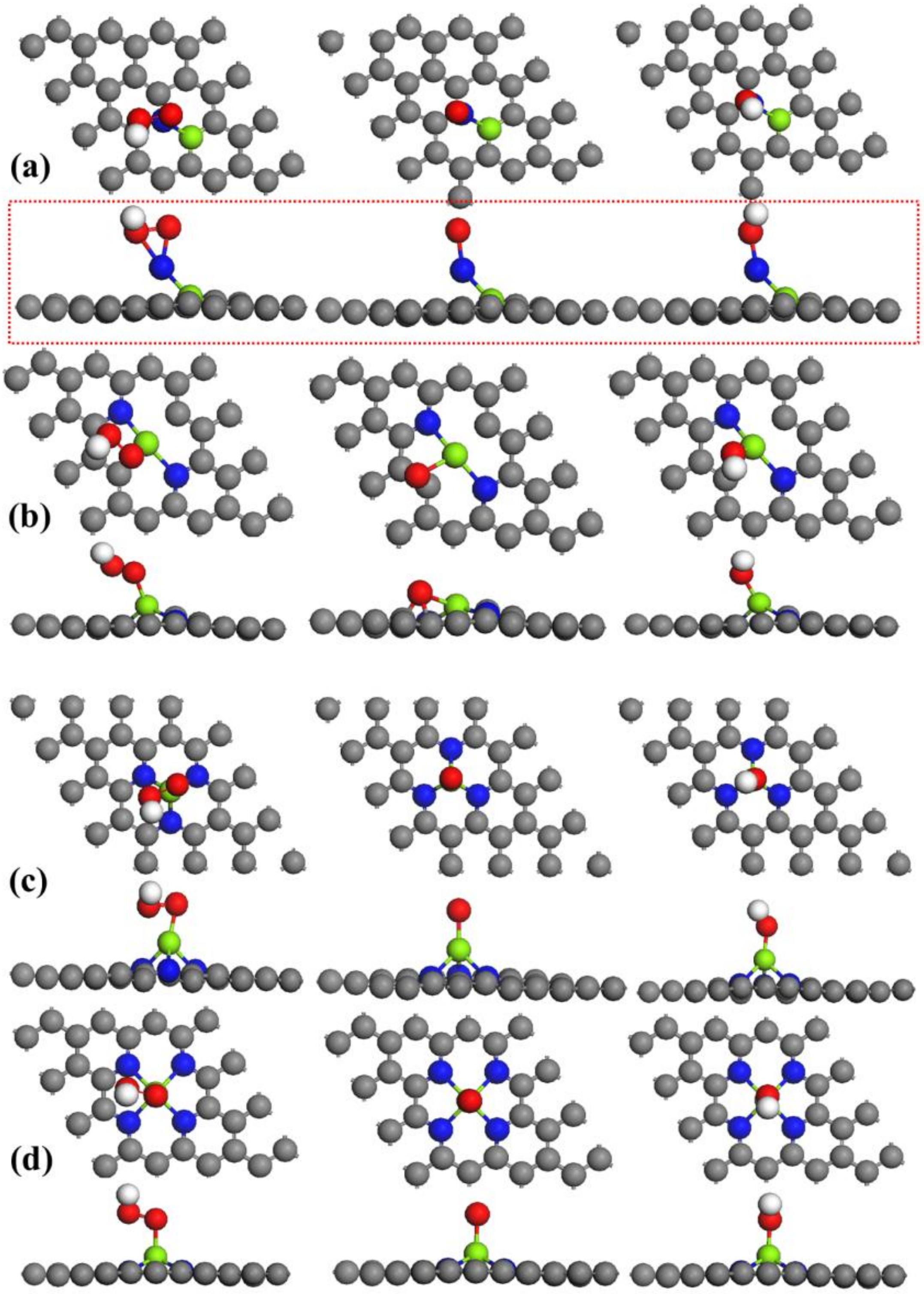

Supplementary Figure 4. Atomistic structure of the intermediates. Transition OOH* state (left panel), O* state (middle panel) and OH* state (right panel) for ORR reaction on (a) MN1C, (b) MN2C, (c) MN3C and (d) MN4C. Green, blue, red, white and gray balls represent Mg, N, O, H and C atoms, respectively. As shown in Figure a, the MN1C

is unstable for ORR process as the metal center get out of plane after interaction with intermediates.

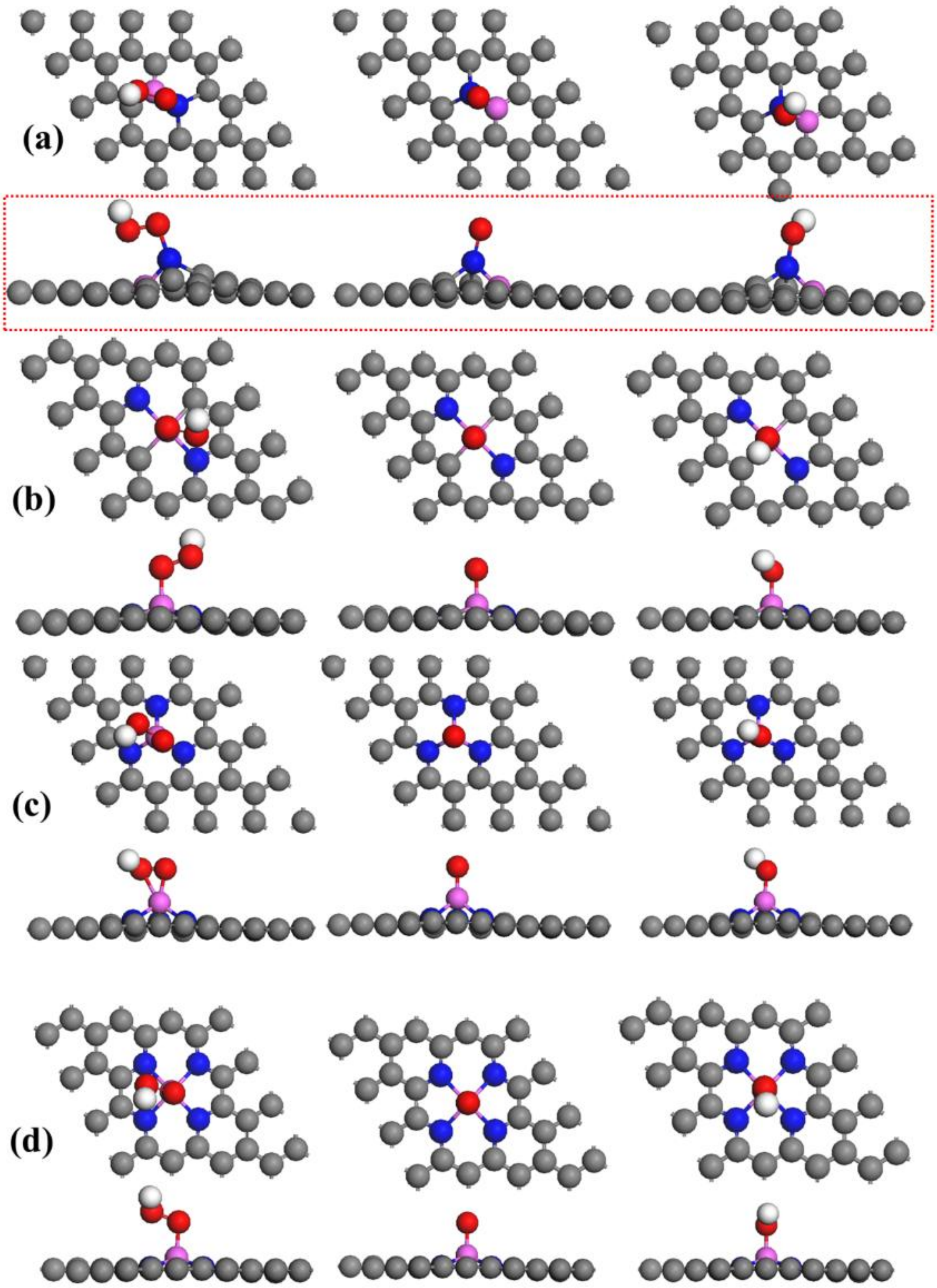

Supplementary Figure 5. Atomistic structure of the intermediates. Transition OOH* state (left panel), O* state (middle panel) and OH* state (right panel) for ORR reaction

on (a) AN1C, (b) AN2C, (c) AN3C and (d) AN4C. Green, blue, red, white and gray balls represent Al, N, O, H and C atoms, respectively. As shown in Figure a, the AN1C is unstable for ORR process as the metal center get out of plane after interaction with intermediates.

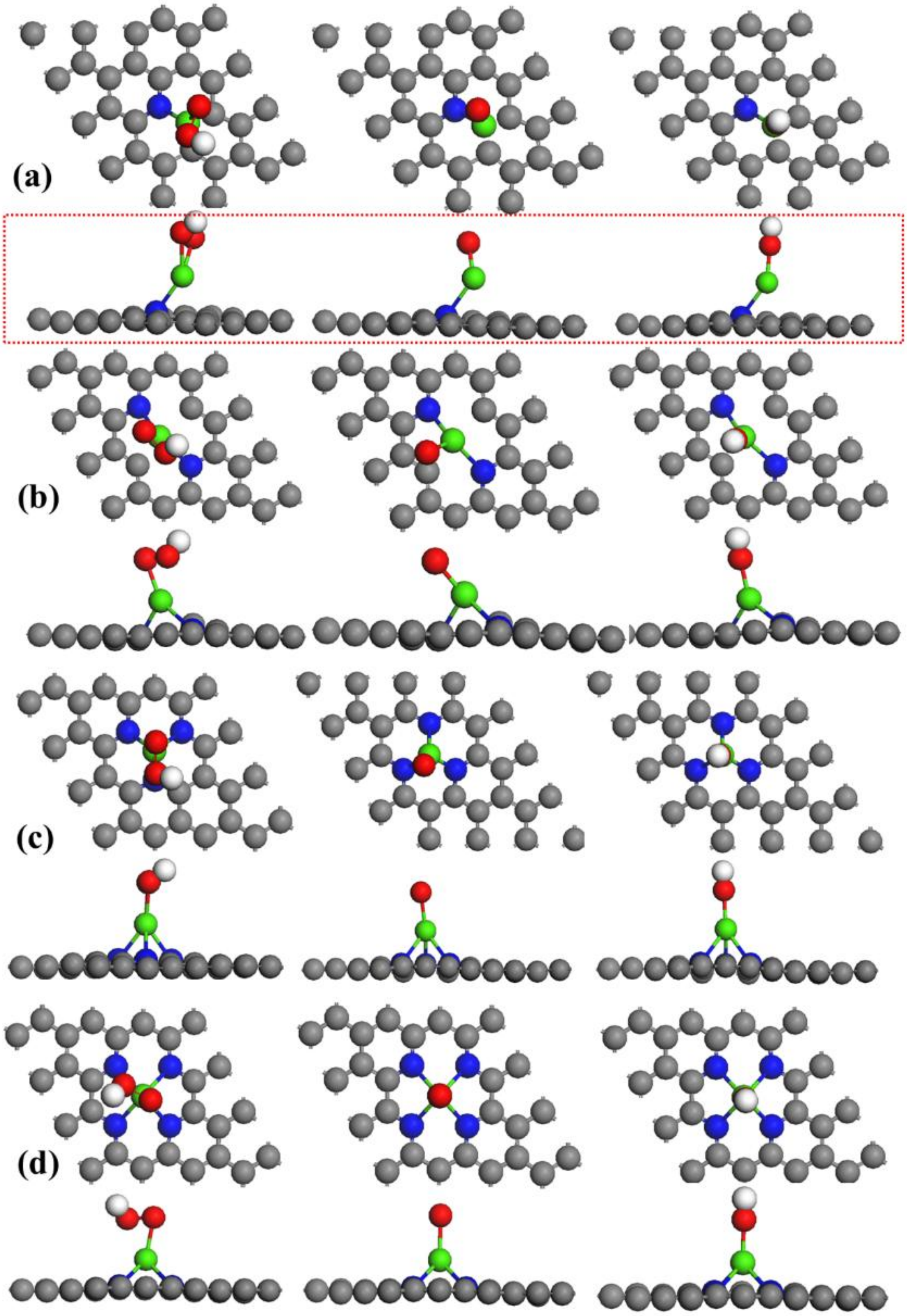

Supplementary Figure 6. Atomistic structure of the intermediates. Transition OOH* state (left panel), O* state (middle panel) and OH* state (right panel) for ORR reaction on (a) CN1C, (b) CN2C, (c) CN3C and (d) CN4C. Green, blue, red, white and gray balls represent Ca, N, O, H and C atoms, respectively. As shown in Figure a, the CN1C

is unstable for ORR process as the metal center get out of plane after interaction with intermediates.

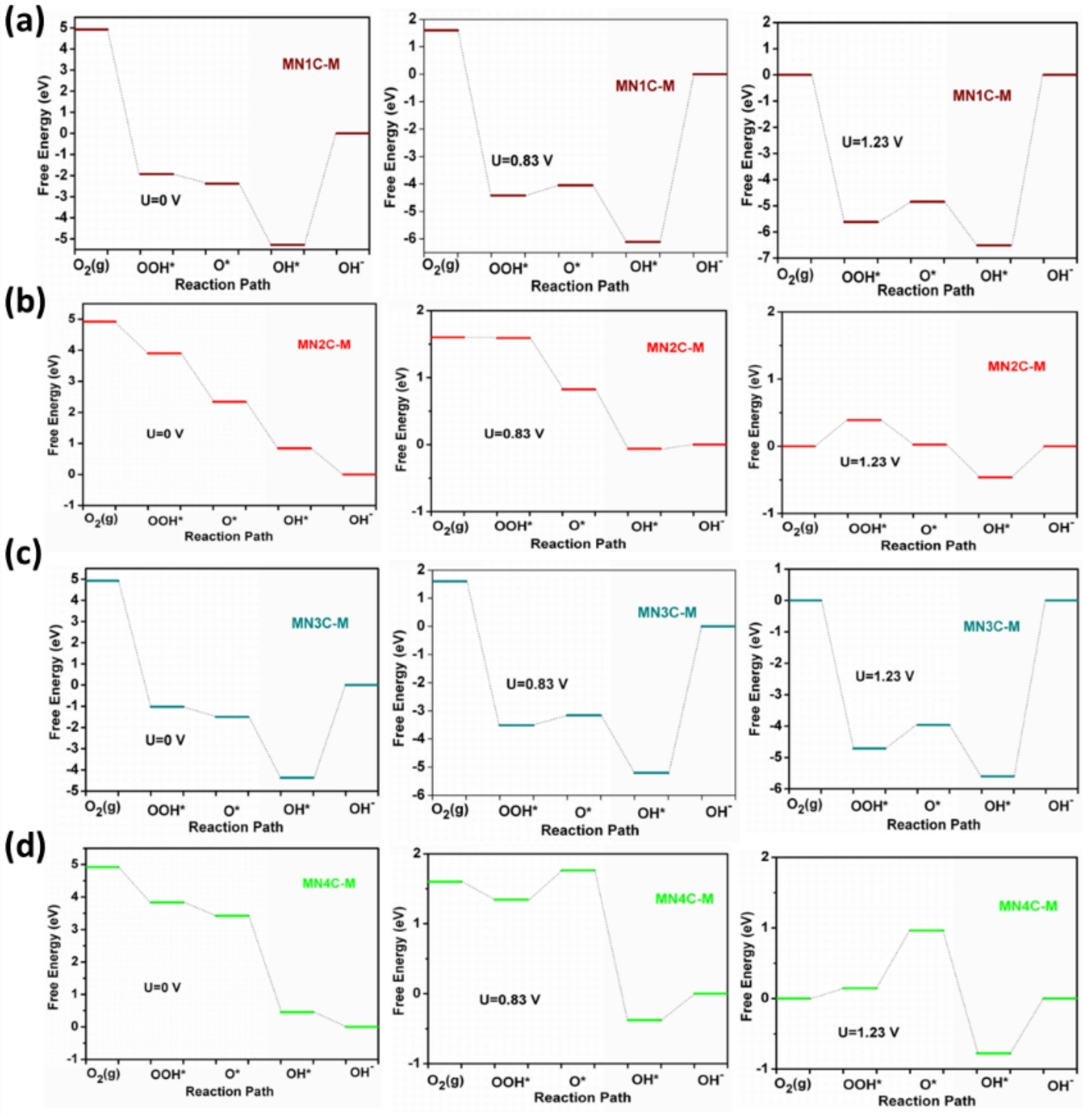

Supplementary Figure 7. Free-energy evolution diagram for ORR. The diagrams for (a) MN1C, (b) MN2C, (c) MN3C and (d) MN4C model through a 4e- associative pathway under electrode potential of U=0, U=0.83V and U=1.23V. And the MNxC-**M** represents the initial active sites are set at metal center (Mg) which is distinguished from other initial sites calculated in section 1.4 in SI.

For Mg sites in ORR, they show downhill trend in oxygen activation step (O_2 transforms to OOH) which reveals Mg sites accelerate the ORR in initial step. However, they show uphill energy barriers from OH release step which hampers the whole process. The MN2C-M with reasonable surface electronic structure shows the smallest

uphill energy barrier of OH release step which has the best activity among MNxC models (Table S4), and MN1C shows the extremely large energy barrier which is caused by unstable structure as shown in Supplementary Figure 4.

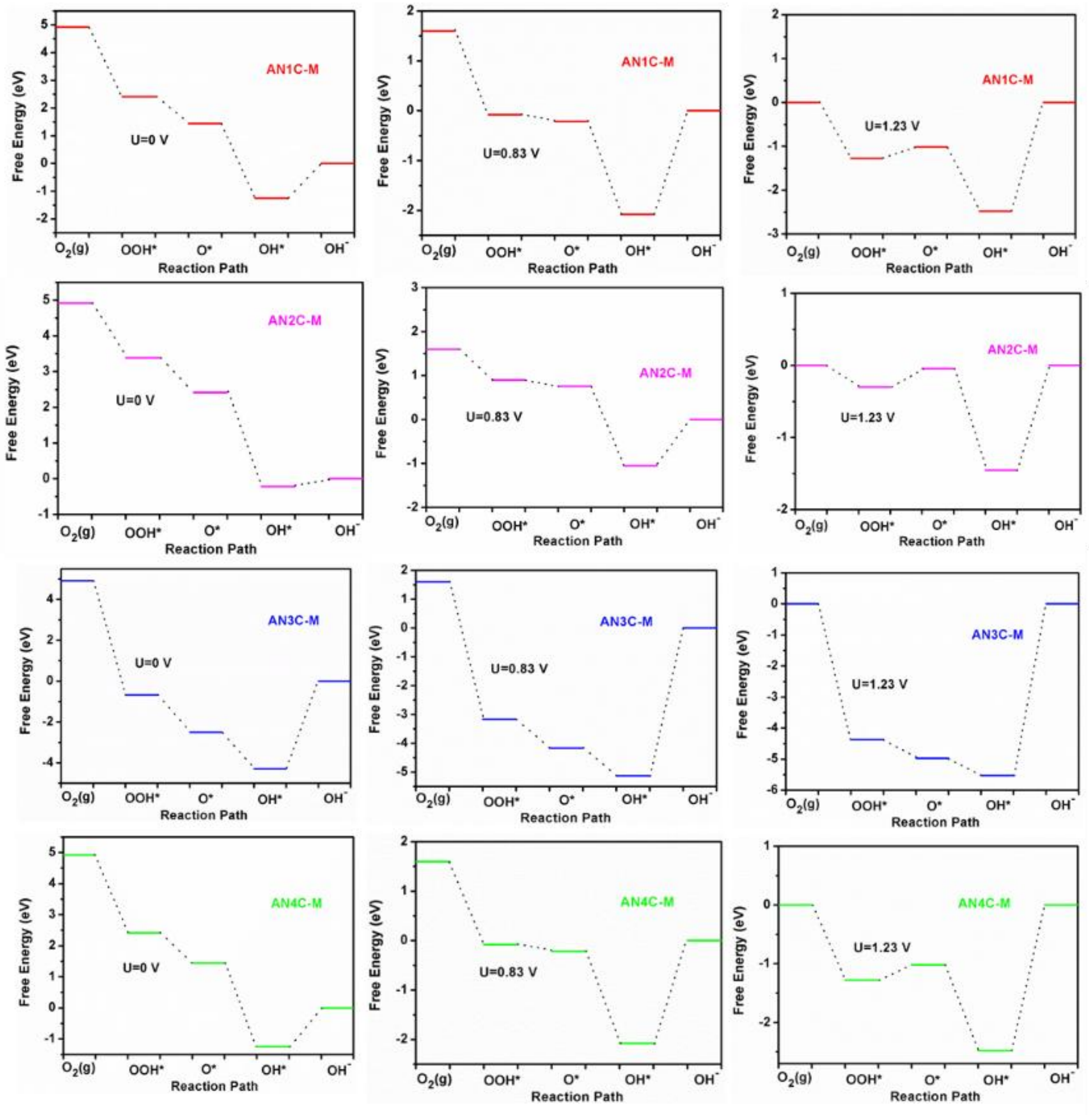

Supplementary Figure 8. Free-energy evolution diagram for ORR of (a) AN1C, (b) AN2C, (c) AN3C and (d) AN4C model through a 4e- associative pathway under electrode potential of U=0, U=0.83V and U=1.23V. The active site is set as the metal center.

For Al sites in ORR, they show downhill trend in oxygen activation step (O_2 transforms to OOH) which reveals Al sites accelerate the ORR in initial step. However, they show larger uphill energy barriers from OH release step than Mg sites which hampers the whole process and lower activity than Mg cofactors.

The Al center is hampered by strong binding of hydroxyl for the large energy barrier of dis-adsorption of OH* in last step. The AN2C-M shows the smallest uphill energy barrier which has the best activity among ANxC models (Table S4), and AN1C shows the extremely large energy barrier which is caused by unstable structure as shown in Fig. S5.

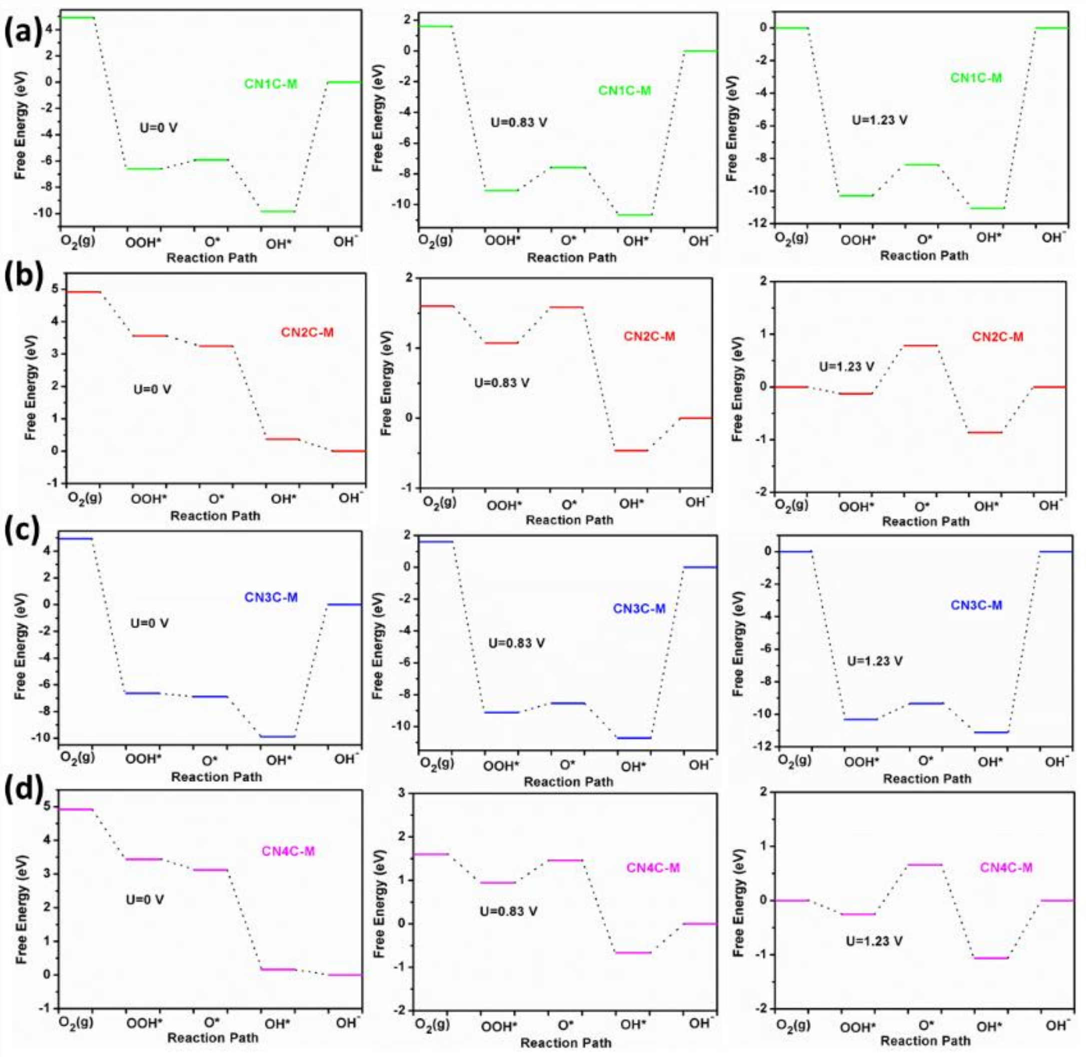

Supplementary Figure 9. Free-energy evolution diagram for ORR of (a) CN1C, (b) CN2C, (c) CN3C and (d) CN4C model through a 4e- associative pathway under electrode potential of U=0, U=0.83V and U=1.23V. The active site is set as the metal center.

For Ca sites in ORR, they show downhill trend in oxygen activation step (O_2 transforms to OOH) which reveals Ca sites are eletrophilic for oxygenated species. However, they show extremely large uphill energy barriers from OH release step which causes bad ORR activity.

The Ca center is hampered by extremely strong binding of hydroxyl for the large energy barrier of dis-adsorption of OH* in last step. The CN2C-M and CN4C-M show closed activity performance among CNxC models (Supplementary Table 4), and CN1C and CN3C shows the extremely large energy barriers which are caused by unstable structure as shown in Supplementary Figure 6.

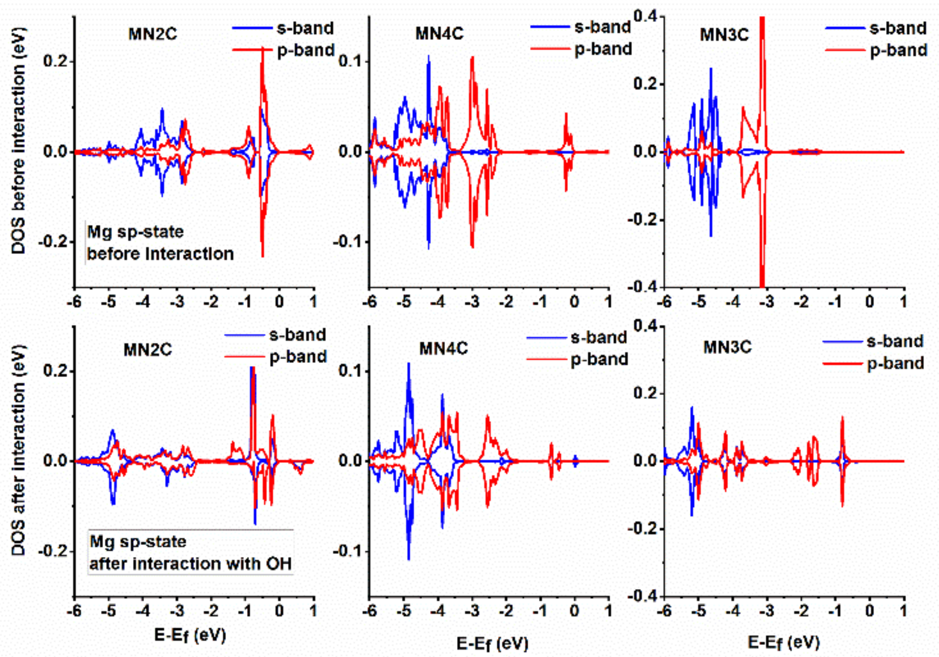

Supplementary Figure 10. The local DOS of Mg before and after interaction with OH. The up row shows the local DOS of Mg in cofactors (MN2C, MN4C and MN3C respectively) before interaction with OH intermediate; the down row shows the local DOS of Mg in cofactors (MN2C, MN4C and MN3C respectively) after interaction with OH intermediate.

It is clearly that p-state of Mg before interaction is closed to Fermi level which would influence the interaction most. After interaction with OH, p-state of Mg are renormalized and some of p-state level (-1 eV to Fermi level) are consistent with broadened 1π state of OH adsorption which reveals that electrons donation from OH occupied the empty hybridized p-state of Mg.

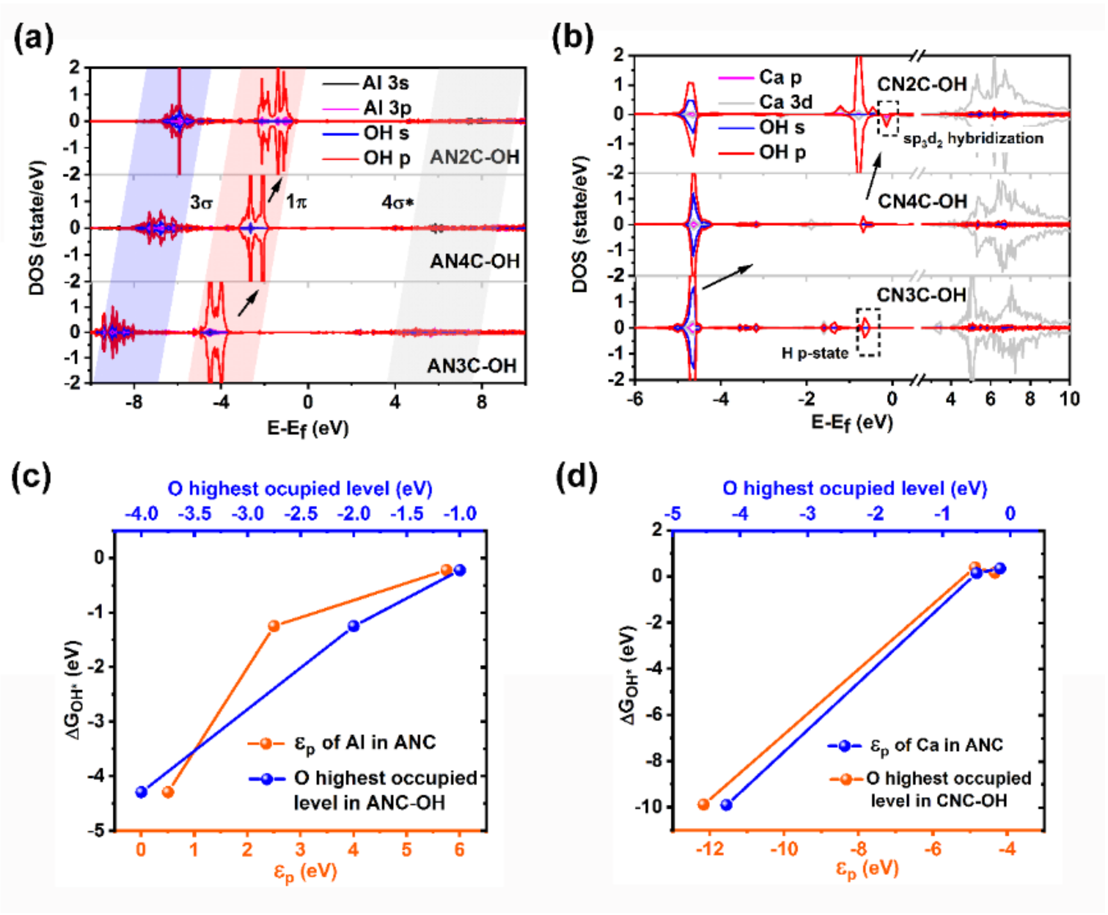

Supplementary Figure 11. The relationship between adsorbate strength of OH* and p-electron feature. (a) Projected DOS of OH adsorption at ANxC. (b) Projected DOS of OH adsorption at CNxC. The 3d empty orbitals may participate CNxC hybridization with sp^3d^2 form. (c-d) Adsorption free energies of OH as a function of the ε_p position of metal atoms for Al (c) and Ca (d) cofactors, and also as a function of highest O occupied state of hydroxyl after interaction. Lines are used to fit the linear relationship.

As shown in **Supplementary Figure** 11a, when OH molecule orbital interacted with p-state of Al surface, they are down shifted and broadened, especially the 3σ (from H 1s-O $2p_z$) and 1π (from lone pair O $2p_{x,\ y}$ electrons) of OH molecule orbitals, which would mainly influence the adsorption strength. As ε_p of Al shifts up, the down shifted degree of 3σ, 1π and $4\sigma^*$ of OH molecule orbital are weakened, in other words, slightly up shifted due to the interaction between up shifted p-state and OH adsorption. The $4\sigma^*$ is away from Fermi level which is negligible to influence adsorption strength. As one can see the black arrows tagging the highest O occupied p-state, AN2C-OH with the highest O occupied p-state processes the weakest adsorption as tabulated in Figure 3c with sky-blue dots.

Similar trend is found in CNxC-OH, while empty d-state at Ca atoms may influence OH adsorption by participation of hybridization of CNxC.

1.2 Characterization of pyrolysis products and ORR performance of the catalysts

The crystal information about Mg-HMT is given in Supplementary Table 7 and Supplementary Figure 12. And the X-ray diffraction pattern (XRD) with simulation (Supplementary Figure 13) and the further Fourier transform infrared (FTIR) confirm the coordination of Mg-HMT (Supplementary Figure 14). The scanning electron microscopy (SEM) images of the precursors are shown in Supplementary Figure 15.

After heat treatment, most Mg evaporated and ligand decomposed to form the N-doped carbon matrix (see thermogravimetry (TG) curves in Supplementary Figure 16). As there is no pyrolysis product for the pure ligands, Mg probably constitutes the carbon framework in pyrolysis of Mg-HMT. Transmission electron microscopy (TEM) and high resolution transmission electron microscopy (HRTEM) tests (Supplementary Figure 17) show there are no metal nanoparticles or oxides detected in catalyst obtained before and after pickling (also confirmed by XRD in Supplementary Figure 17d). Note that, as shown in Supplementary Figure 18, due to the large scale of material, the graphite (002) peak could be observed if it was not fully ground. Although there is no signal about the Mg-oxides in the XRD and TEM results, further pickling is taken to get rid of the inactive residual not detected. And the FTIR result (Supplementary Figure 19) points out slight Mg-O stretching vibrations disappeared after pickling, which reveals that some tiny amorphous Mg-oxide clusters are removed and they are not active substance proved by the enhanced electrochemical performance later. And FTIR result also reveals nitrogen doping by C-N stretching vibration in Mg-N-C. The Brunauer-Emmett-Teller (BET) adsorption–desorption isotherms as shown in Supplementary Figure 20 shows the larger surface area and the increasing pore with diameter under 2nm which may be induced by the removal of oxygenated species. The high resolution transmission electron microscopy (HRTEM) image of Mg-N-C (Figure 3b) confirms the porous graphitic carbon in the absence of impurity. It seems no change after pickling in XRD (Supplementary Figure 17d) where the broad peak at about $20-30^{\circ}$ indexes the (002) plane of the low degree graphitized carbon and no diffraction peak of metal or oxides is observed. Raman spectra in Supplementary Figure 17e confirm that the catalysts are composed of graphene analogue doped by a large amount of heteroatoms (high ratio of D and G) [1,2] and no change after pickling.

For the XPS results, we referred the binding energy position from the NIST databases and the reported references. From NIST databases, the Mg 1s and 2p spectrum in Supplementary Figure 22 a and b clearly show that no metallic Mg signal is found (the positions of 1303-1303.5eV for Mg 1s and 49.3-49.7eV for Mg 2p) and

there is a +2 oxidation state of Mg in the catalyst. And the Mg-O bond position is out of range (the positions of 1303.9eV for Mg 1s and 50.25eV for Mg 2p). As shown below in Supplementary Figure 22a, the Mg 1s spectra can be deconvoluted into two peaks, one is Mg-N bond (at 1304.2eV referred to MgPc), another peak located at 1305.2eV matches well with the literature reported Mg-C bond[3]. And also there is clearly two peaks in the Mg 2p spectra which can be deconvoluted into Mg-N bond (at 50.6eV referred to MgPc) and Mg-C bond (at 52.6eV[4,5]). The Supplementary Figure 22c shows that there are heteroatoms doped in carbon matrix in the catalyst. The C1s spectra in XPS result (as shown in Supplementary Figure 22a) is deconvoluted into different peaks at 284.7 eV, 285.7 eV, 287.4 eV, and 290.1 eV, which are attributed to C-C/C-H, C-N/C-O, C=O and π^*, respectively (oxygen in carbon framework is due to the in situ decomposition of oxygen-bearing ligand or remnant of air in precursor) [6]. In Figure S22 d, a weak peak exists at ~529.8 eV (lattice oxygen[7,8]) before pickling, and according to FTIR results it is the Mg-O bond. This peak disappeared after acid pickling which matches well with the FTIR results in Supplementary Figure 19 and also indicates that there is no Mg-O bond in the final Mg-N-C catalysts. The spectrum of N 1s in Figure 3g can be deconvoluted into four peaks at 398.5 eV, 400.1 eV, 400.8 eV and 401.7 eV, corresponding to pyridinic-N, pyrrolic-N, Mg-Nx and graphitic-N, respectively[9], which reveals the N doping forms in Mg-N-C catalyst. In conclusion, XPS result confirms the existence of Mg, N co-doped in carbon based-catalyst, rather than metallic Mg.

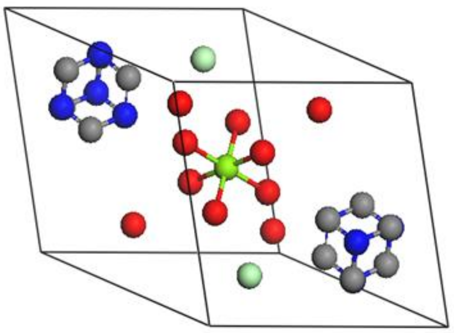

Supplementary Figure 12. The atomistic configuration of Mg-HMT cell. The structure is similar to that of hexaaquacobalt (II) dichloride bis (hexamethylenetetramine) tetrahydrate (Co-HMT) (Data can get from The Cambridge Crystallographic Data Centre (CCDC), CCDC number: 1184231, and Inorganic Crystal Structure Database (ICSD), ICSD number: 159012).

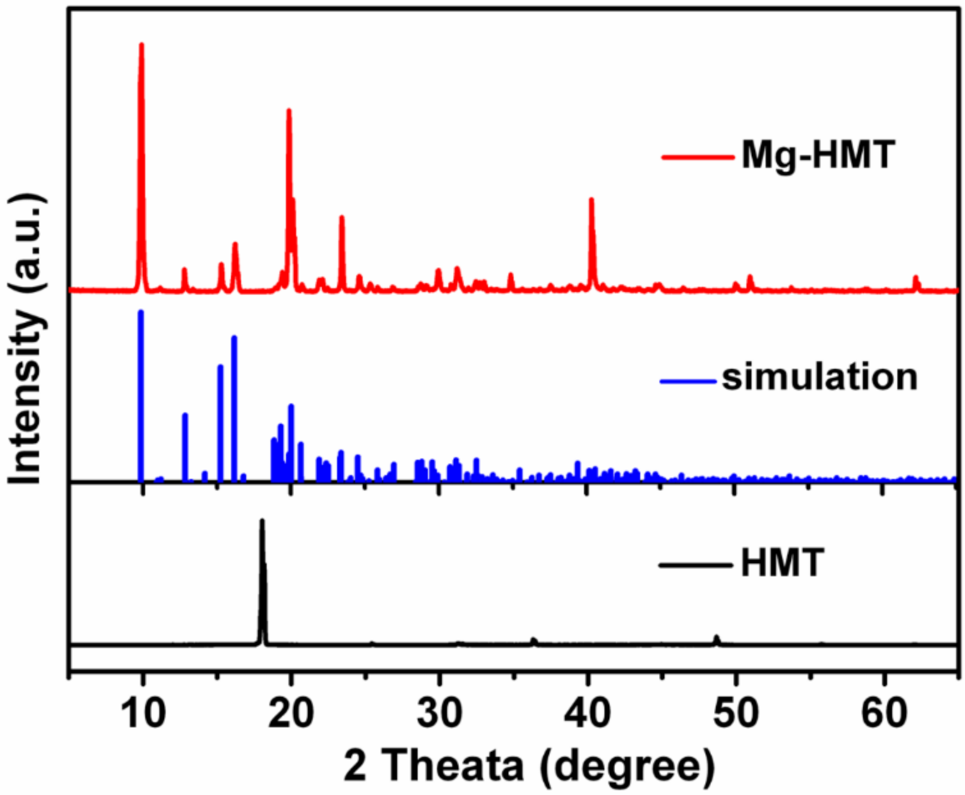

Supplementary Figure 13. The XRD patterns of Mg-HMT precursor. The low degree peaks of Mg-HMT match well with the XRD simulation results and are distinct from that of HMT, which reveals that the coordination from metal ions and ligand in Mg-HMT.

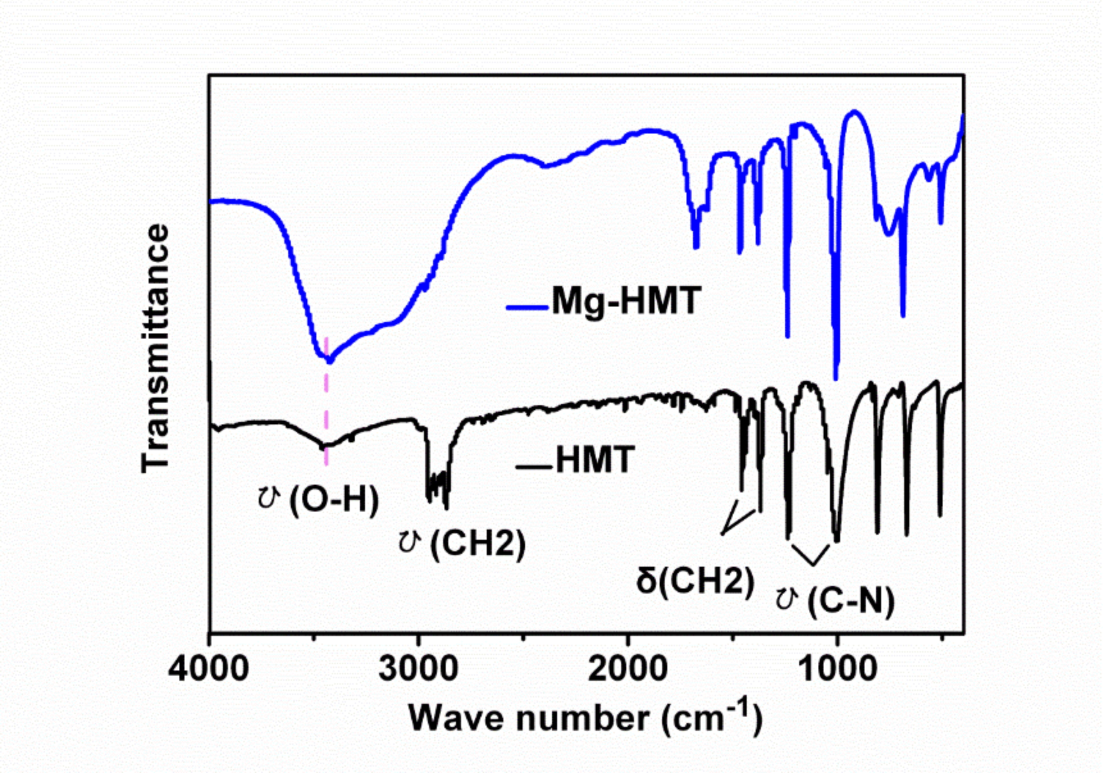

Supplementary Figure 14. The FTIR analyses for Mg-HMT precursor. The vibration at ~2900 cm^{-1} in HMT disappears while a new enhanced vibration is observed at ~3500 cm^{-1} confirms the coordination.

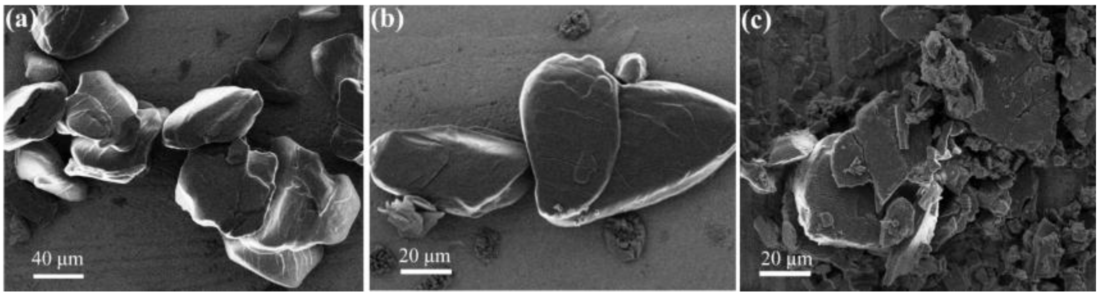

Supplementary Figure 15. The SEM images for Mg-HMT precursors. Particles are shown with different sizes after drying.

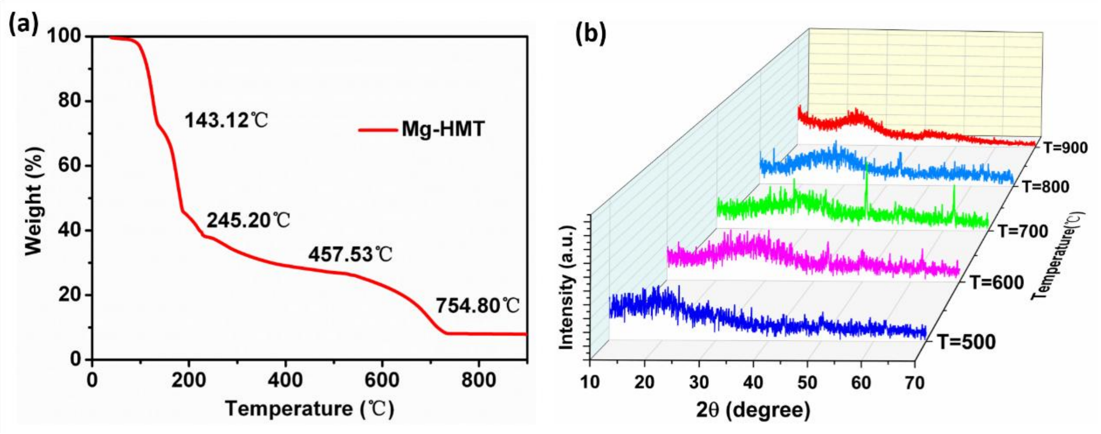

Supplementary Figure 16. Analysis of the formation of Mg-N-C. (a) Thermogravimetry curve of Mg-HMT precursor and (b) corresponding XRD patterns.

The melting point and boiling point of metal Mg is 651 ℃ and 1108 ℃, nano-metal particles could be much lower due to the high surface area.

We had synthesized the pyrolysis of precursor Mg-HMT in different temperatures for one hour. And we found the continuous change of pyrolysis as shown in Figure S16. From the TGA curves of pyrolysis in Figure S16 a, there is a weight loss in temperature range 500-700 ℃. According to the Figure S16b, the precursor is not completely change to carbon in 500 ℃, until 600 ℃ there is a broad peak at 26-28 °in XRD, and meantime there is slight signal at 42.8 °and 62.2 °which represent the existence of MgO at this temperature. In temperature 700 ℃, the signal of MgO is stronger than that of in temperature 600 ℃, which reveals the MgO amount is become more. While it is weaker in temperature 800 ℃ and is almost absence in temperature 900 ℃.

It is interesting and we had referred the references. It had been reported that the MgO can be reduced by carbon[10,11] and especially it can be occurred at 900 ℃[10], so we think the MgO is dioxide by carbon and transferred to Mg and CO[10] in pyrolysis, and we found the quartz crucible is polluted so that it turned to be gray with metallic luster after heat treatment in temperature 900°C. Although XRD patterns didn't give a apparently signal of MgO in pyrolysis in temperature 900 ℃ before acidic etching, XPS still showed a weak signal of lattice oxygen referred to Mg-O bond as shown in Figure S22 d, so these results confirms the reduction of MgO by carbon and a little amount MgO undetected maybe existence in the pyrolysis in temperature 900 ℃. It also indicates that the Mg cofactors probably be formed at around temperature 900 ℃ in this reduction process. There are some papers verified this similar transformation process, like FeO_x is transferred to Fe-N coordination in the pyrolysis[12–14].

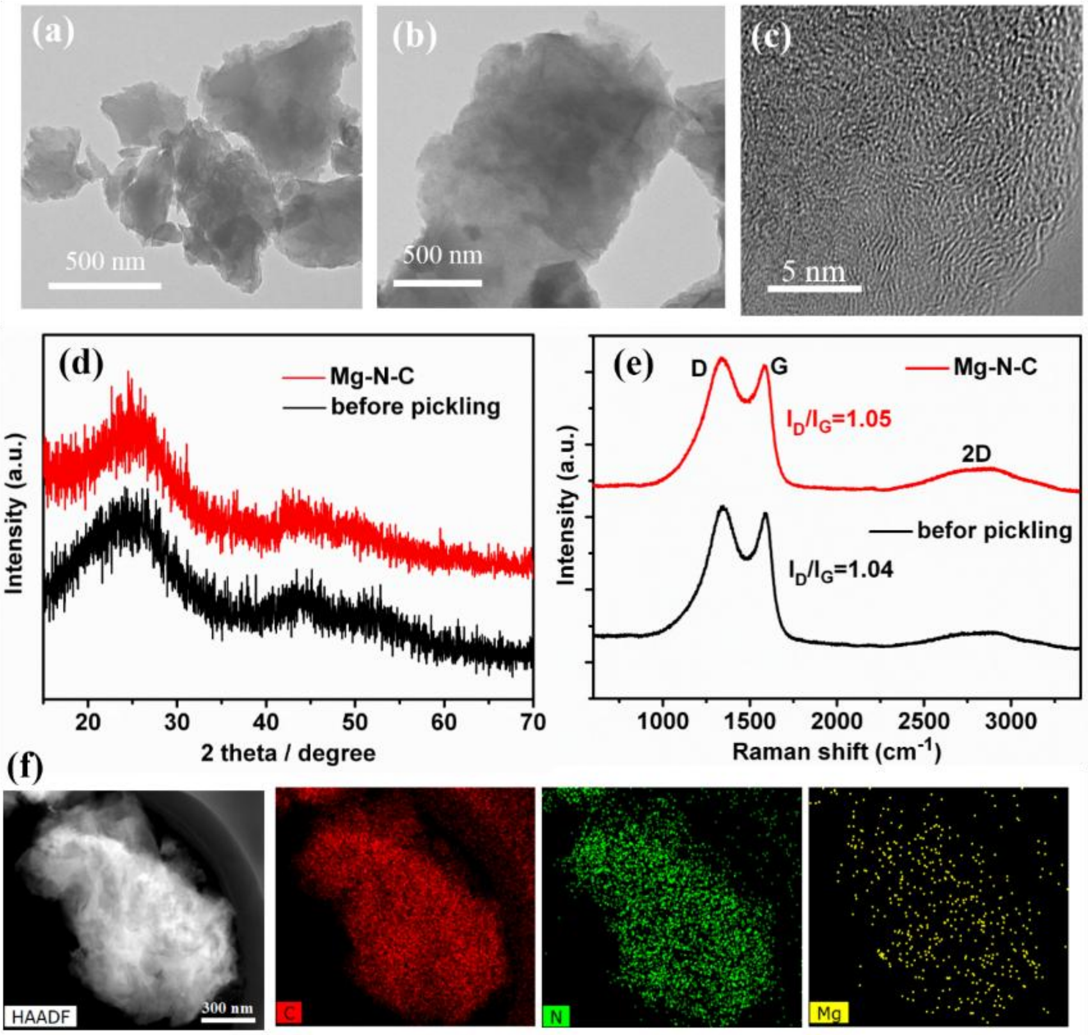

Supplementary Figure 17. The morphology and structural characterization. (a-b) The TEM images about the catalysts before and after acid pickling. (c) HRTEM image of the graphitized carbon Mg-N-C before pickling. No metal nanoparticles or oxide is observed. (d-e) The XRD patterns and the Raman spectrum for the catalysts before and after etching treatment. There is no obvious change. (f) The Energy dispersive X-ray spectroscopy (EDS) images about the elements distribution of C, N and Mg in Mg-N-C catalysts.

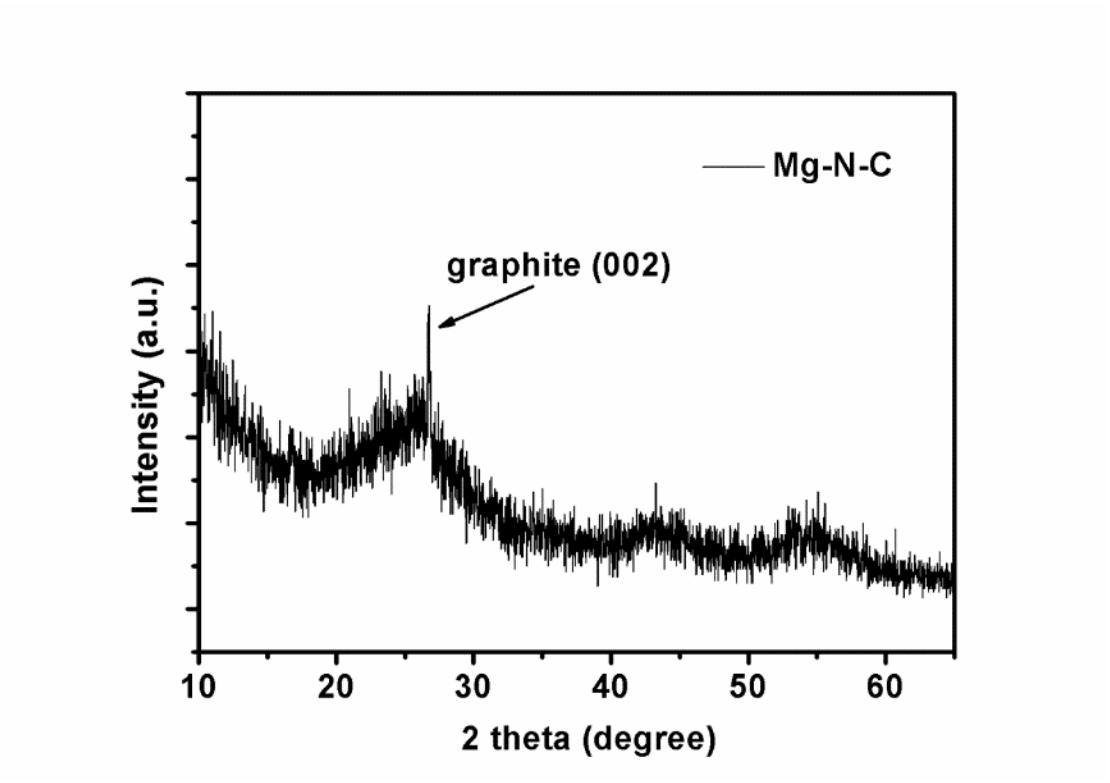

Supplementary Figure 18. XRD pattern of Mg-N-C with large particles. Graphite (002) peak [15] is observed since the products are not fully ground.

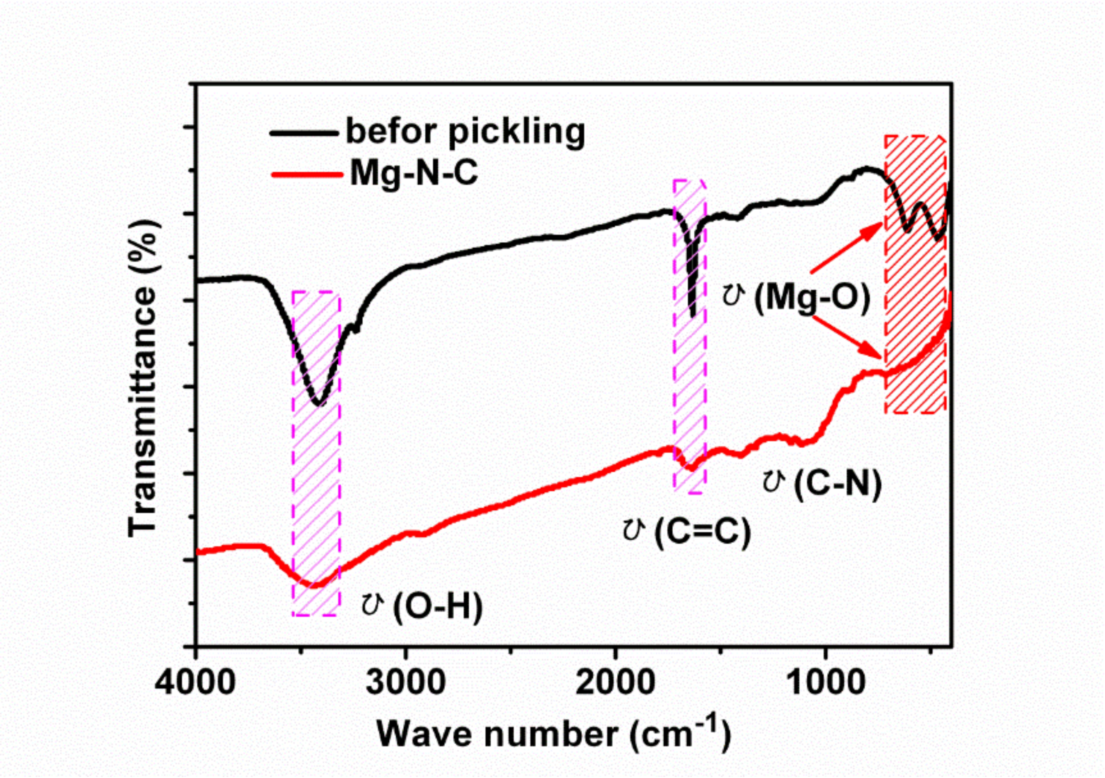

Supplementary Figure 19. FTIR analysis for the pyrolysis products. The peak at ~500 cm^{-1} [16] in the product before pickling disappears in the curve for Mg-N-C, which indicates that no Mg-O exists after pickling.

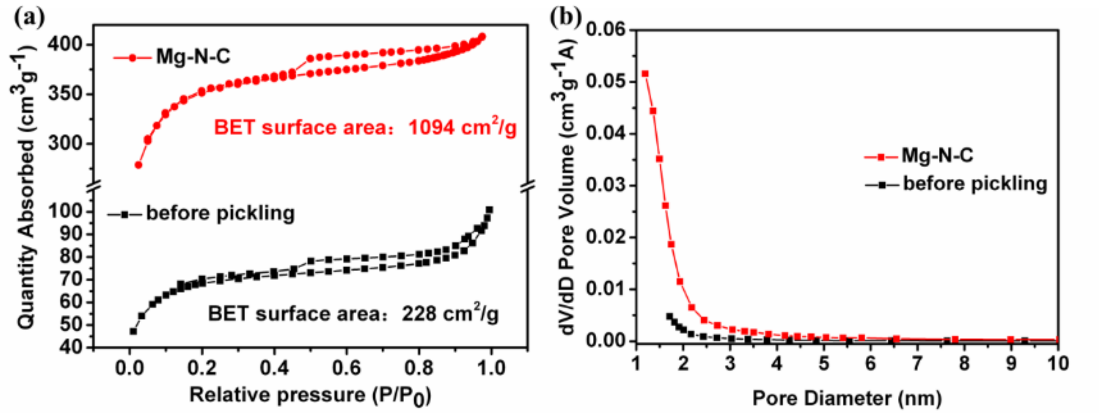

Supplementary Figure 20. The BET adsorption–desorption isotherms for products before and after pickling. The larger surface may expose more active sites after pickling and improve the ORR performance.

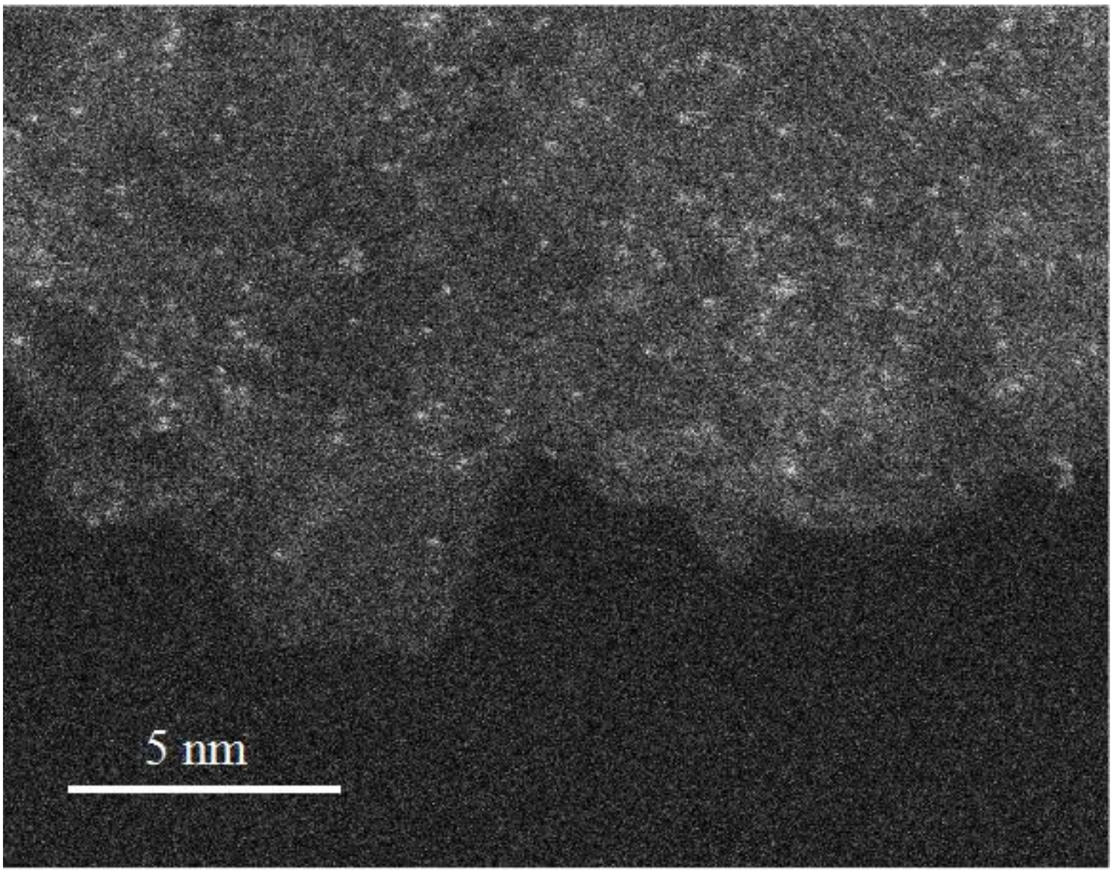

Supplementary Figure 21. HAADF-STEM image of Mg-N-C in large scale. The bright dots are isolated Mg atoms in graphitic carbon matrix.

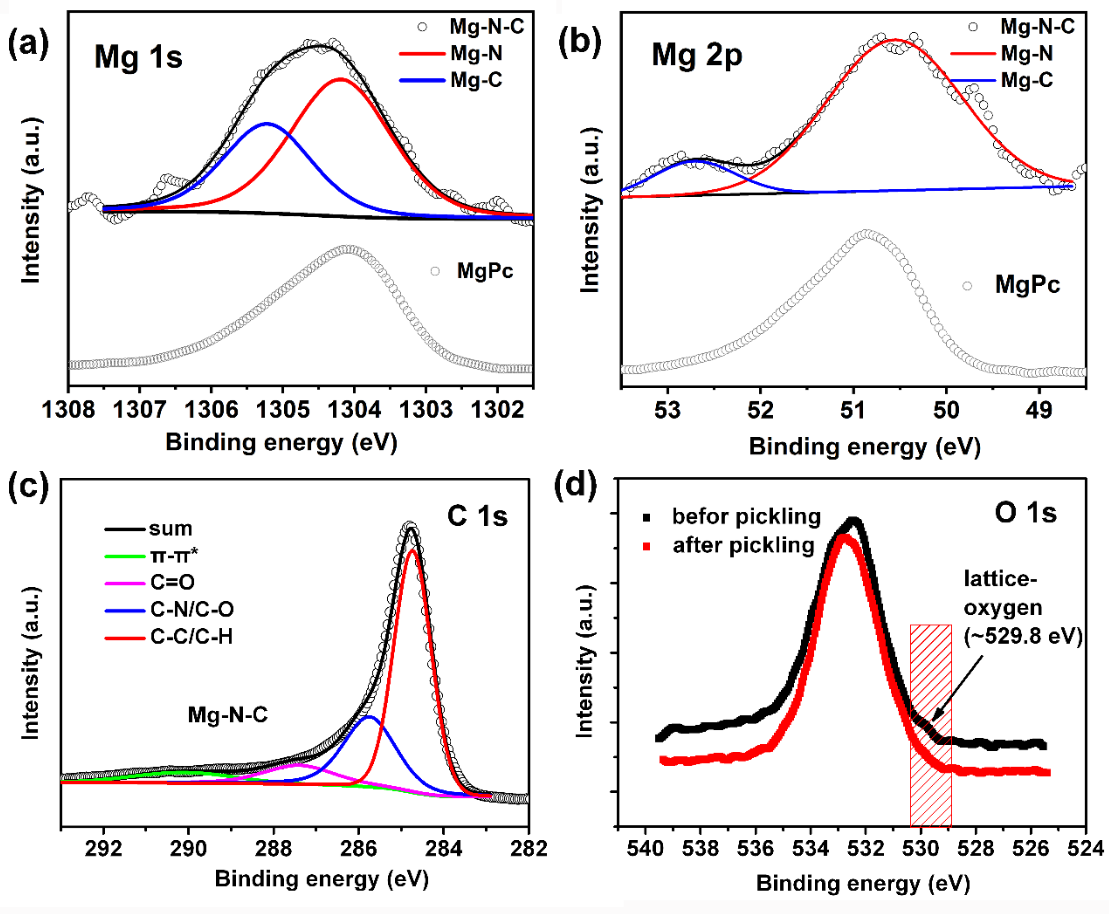

Supplementary Figure 22. The valence state of elements in Mg-N-C catalyst from XPS. (a and b) Mg 1s and 2p spectra in Mg-N-C and Mg-Pc as referred. (c) C 1s spectrum. (d) The O 1s signal from XPS before and after pickling.

For the XPS results, we referred the binding energy position from the NIST databases and the reported references. From NIST databases, the Mg 1s and 2p spectrum in Figure a and b clearly show that no metallic Mg signal is found (the positions of 1303-1303.5eV for Mg 1s and 49.3-49.7eV for Mg 2p) and there is a +2 oxidation state of Mg in the catalyst. And the Mg-O bond position is out of range (the positions of 1303.9eV for Mg 1s and 50.25eV for Mg 2p). As shown below in Supplementary Figure **22**a, the Mg 1s spectra can be deconvoluted into two peaks, one is Mg-N bond (at 1304.2eV referred to MgPc), another peak located at 1305.2eV matches well with the literature reported Mg-C bond[3]. And also there is clearly two peaks in the Mg 2p spectra which can be deconvoluted into Mg-N bond (at 50.6eV referred to MgPc) and Mg-C bond (at 52.6eV[4,5]). The Figure c shows that there are heteroatoms doped in carbon matrix in the catalyst. In Figure d, a weak peak exists at ~529.8 eV (lattice oxygen[7,8]) before pickling, and according to FTIR results it is the

Mg-O bond. This peak disappeared after acid pickling which matches well with the FTIR results in Supplementary Figure 19 and also indicates that there is no Mg-O bond in the final Mg-N-C catalysts.

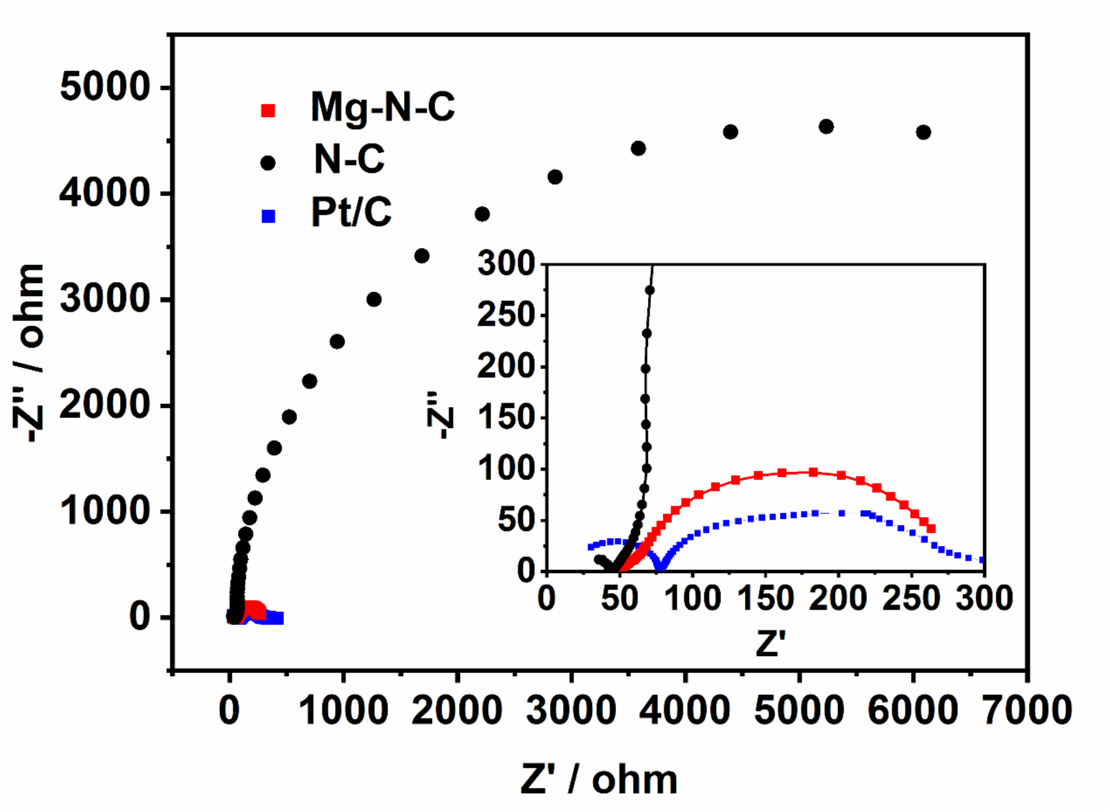

Supplementary Figure 23. Electrochemical impedance spectroscopy (EIS) of Mg-N-C. Insert is the larger scale of EIS. Mg-N-C has much lower charger transfer resistance than that of N-C and is close to Pt/C which contributes faster electrons transfer at catalyst surface.

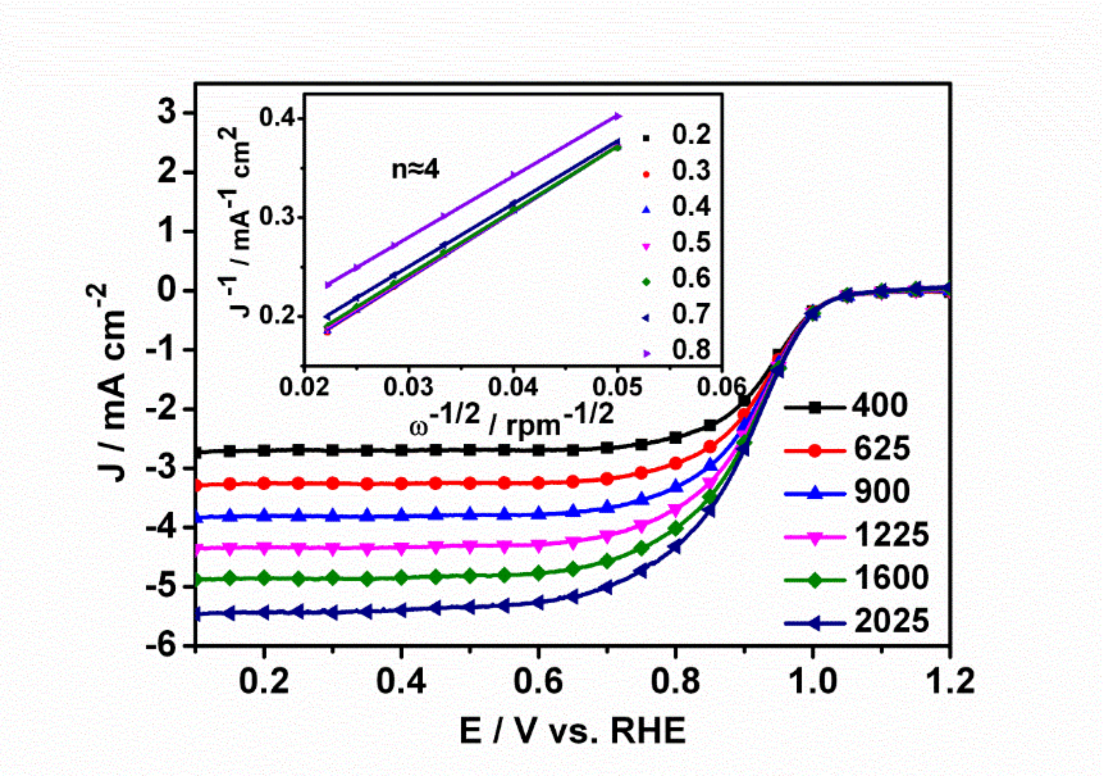

Supplementary Figure 24. The dependence of LSV on different rotation rates for Mg-N-C catalyst during the RDE test (the label for different curves is rpm). The insert shows the electrons transfer number calculated according to the the K-L equation (the label for different curves is V).

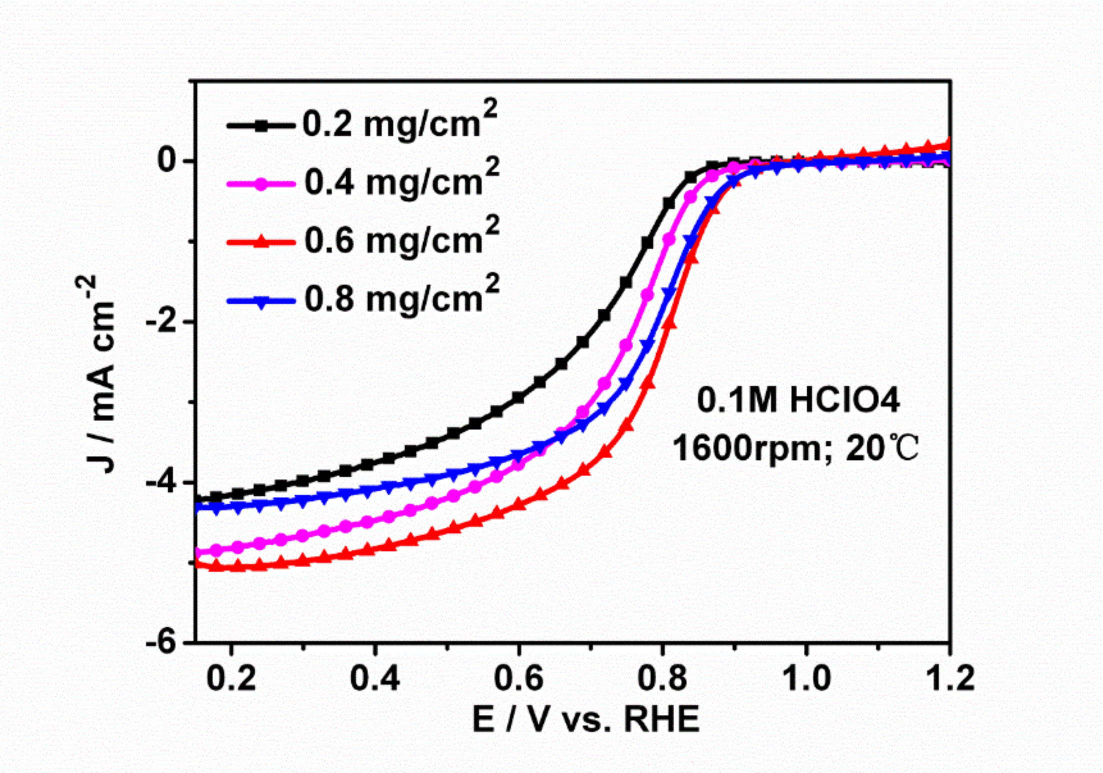

Supplementary Figure 25. The loading dependence of $E_{1/2}$ of Mg-N-C with 1600 rpm in 0.1M HClO4 solution.

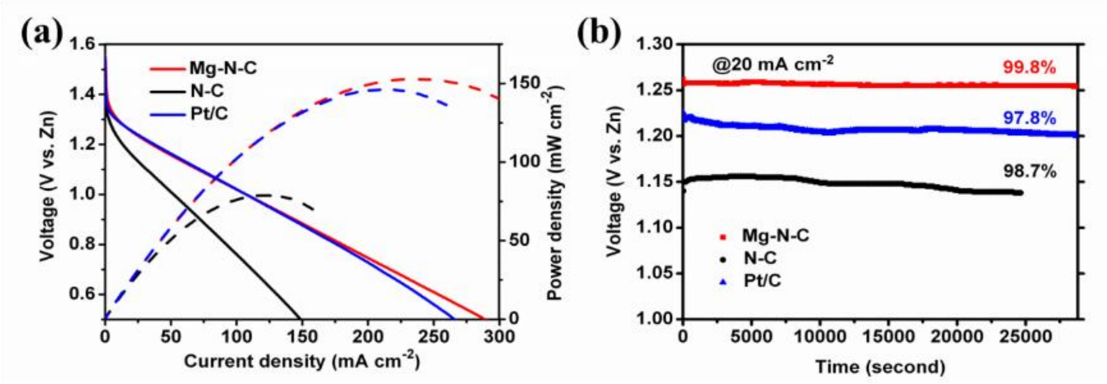

Supplementary Figure 26. The comparison of zinc–air battery performance of Mg-N-C with N-C and Pt/C catalysts. (a) The power density of catalysts. (b) Constant current discharge test. The Mg-N-C shows better performance than Pt/C and excellent stability over time.

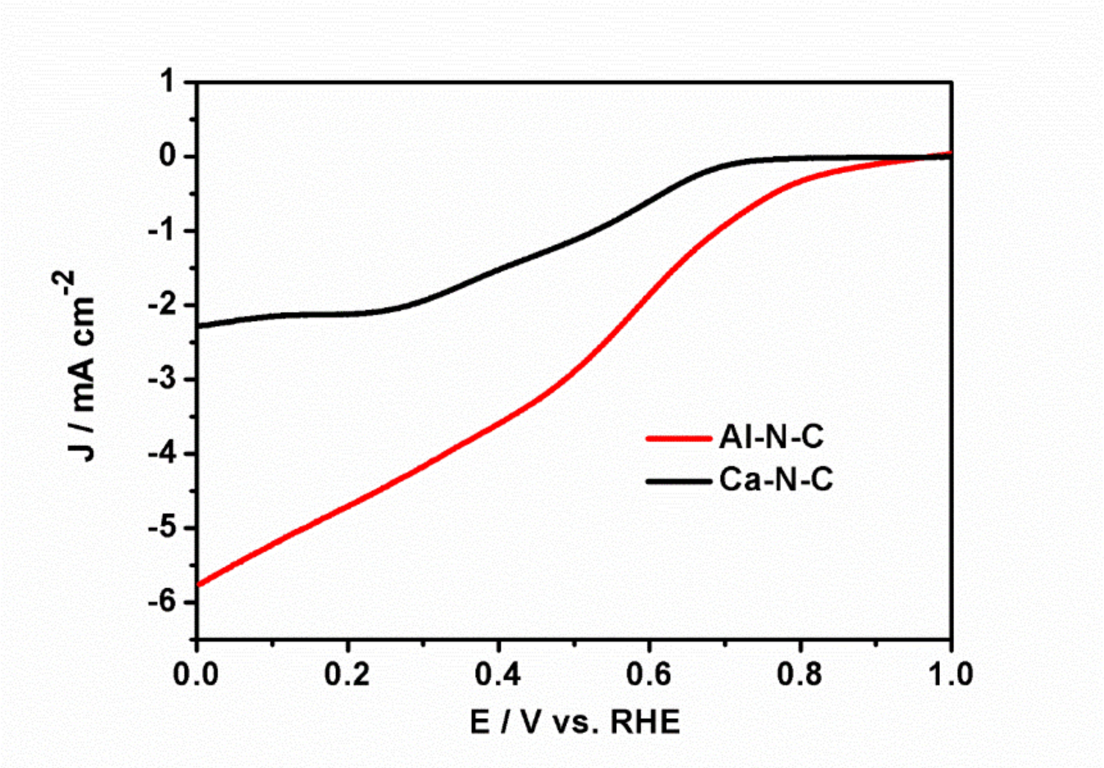

Supplementary Figure 27. The ORR performance of Al-N-C and Ca-N-C.

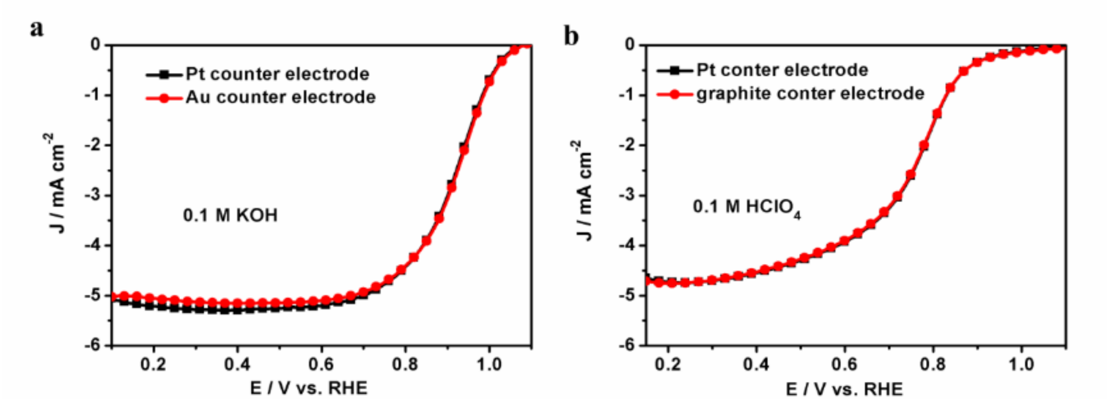

Supplementary Figure 28. The caparison of the influence from different counter electrodes used in (a) basic solution and (b) acidic solution. It clearly shows that there is almost no change when alter the counter electrodes in initial ORR performance test.

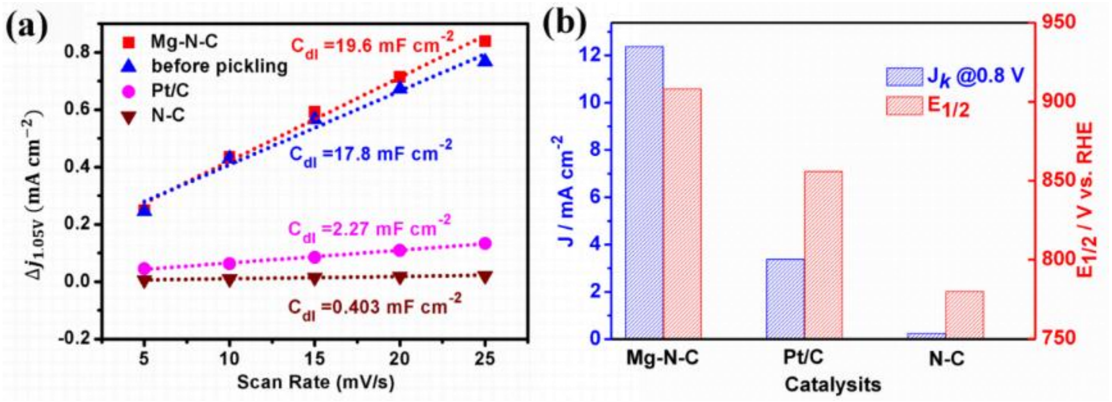

Supplementary Figure 29. Electrochemical evaluation of Mg-N-C. (a) The double layer capacitance (C_{dl}) of catalysts, which is used to represent the ECSA. (b) The kinetic current density (J_k) of catalysts to evaluate the ORR performance.

With higher value of C_{dl}, the ECSA lined with C_{dl} is larger. The Mg-N-C shows the highest value of C_{dl} and so as the product before pickling which indicated the high intrinsic ORR activity of Mg-Nx structure. The larger ECSA of Mg-N-C than catalyst before pickling may be caused by the acid etching which opens the pore structure. Due to the ECSA value from this method is inaccurate (always much higher than real reaction active sites) caused from extra carbon active sites [17,18], the standardized current density from ECSA ($j_{\mathrm{ECSA}} = \frac{j}{\mathrm{ECSA}}$) is underestimated, so we only the standardized current density from geometric area ($j_{\mathrm{Geom}} = \frac{j}{\mathrm{A}_{Geom}}$) to evaluate the performance of catalysts, meanwhile the C_{dl} to represent the ECSA.

Meantime, we also use kinetic current density (J_k) to evaluate the ORR performance of catalysts, as shown in Fig. b, and Mg-N-C shows the highest value up to 12.4 mA cm^{-2}, which is better than 7.2 mA cm^{-2} of Pt/C and 0.4 mA cm^{-2} of N-C.

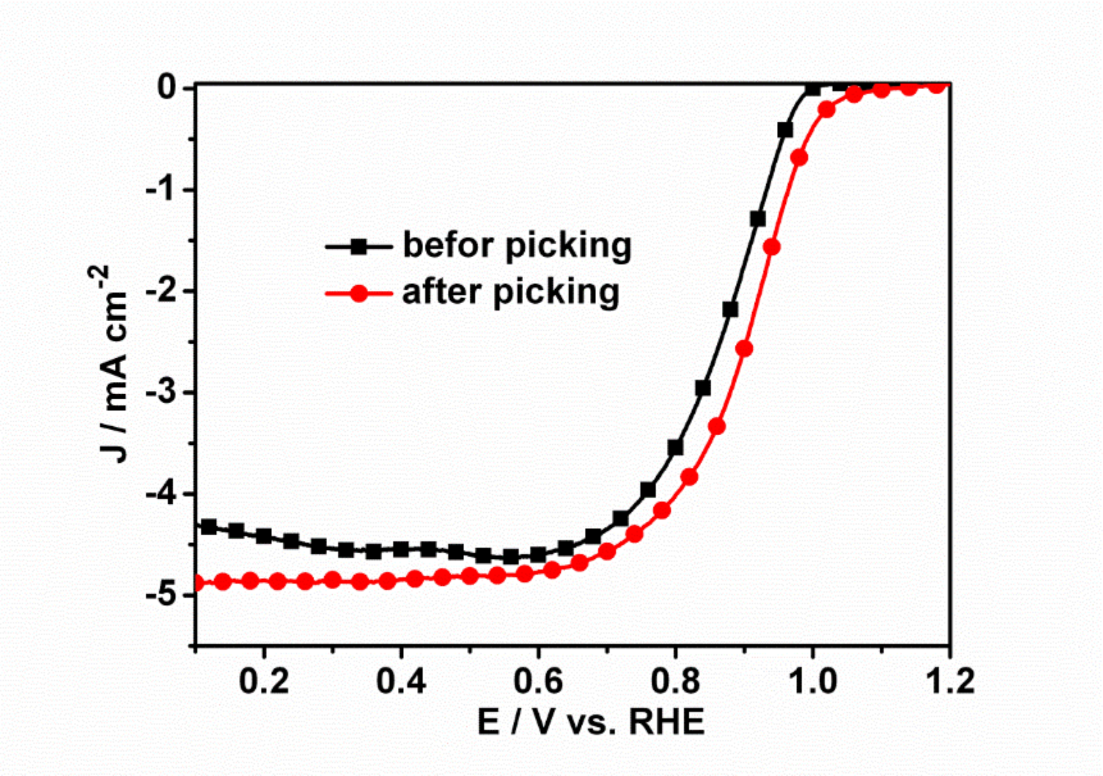

Supplementary Figure 30. The effect of pickling on ORR performance of Mg-N-C catalysts.

After pickling, the LSV curve shows slightly right shift, indicating enhanced catalytic activity. This is caused by larger BET surface area in the products after pickling as illustrated in Supplementary Figure 20.

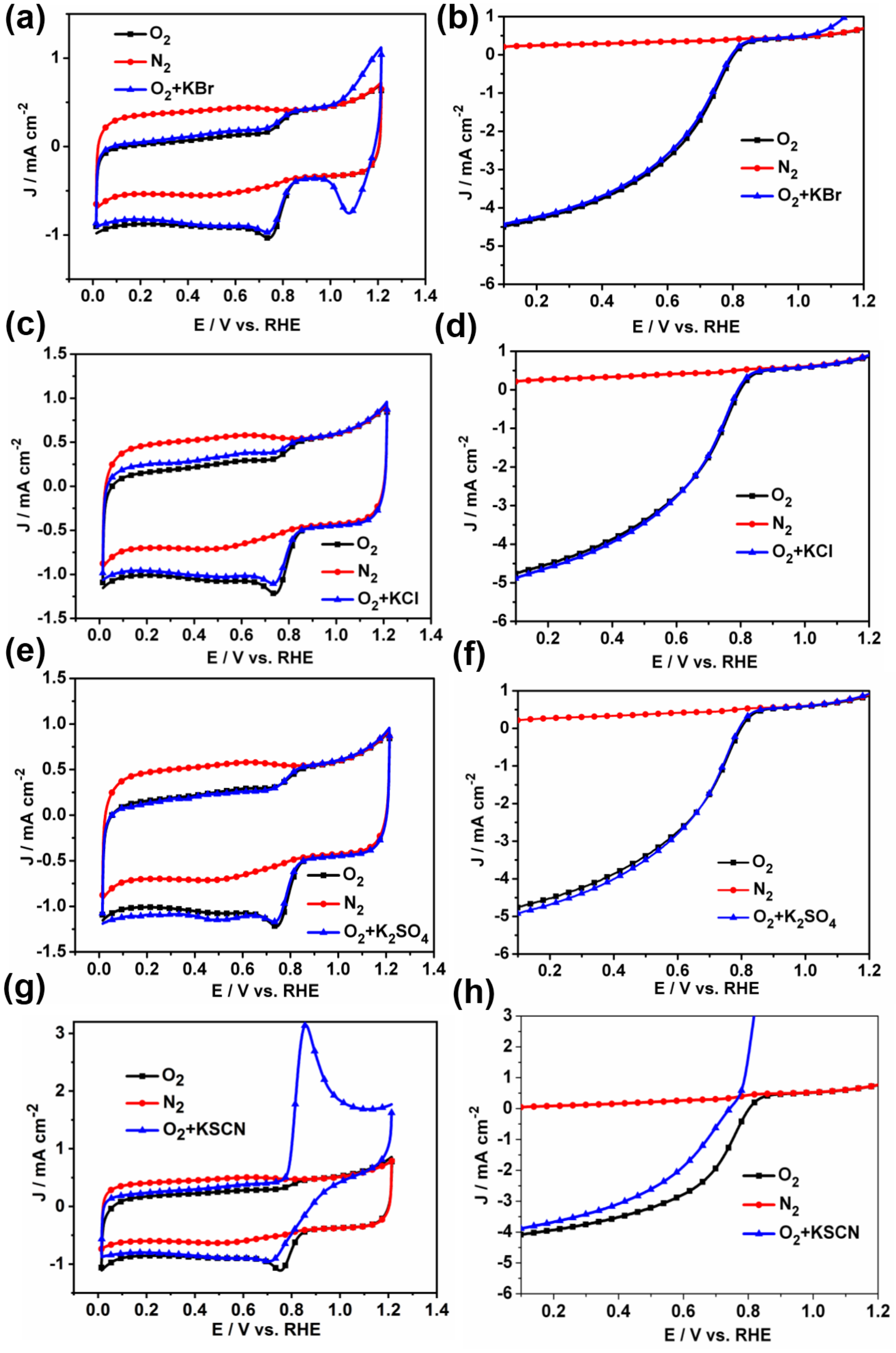

Supplementary Figure 31. Poisoning effect of the Mg-N-C catalyst tested. CV and LSV measurements were carried by adding different anions (0.01M) into the solution. (a-b) Adding KBr. (c-d) Adding KCl. (e-f) Adding K_2SO_4. (g-h) Adding KSCN.

KSCN can block metal sites and poisons the active sites [19–22], as shown in (g and h), after adding 0.01M KSCN, there is a negative shift of 109 mV in $E_{1/2}$, indicating loss of activity. While it still has an $E_{1/2}$ of more than 610mV. This result confirms the activity contribution of metal Mg sites but also inferred the activity contribution of carbon sites[23,24]. Other anions like Br^-, Cl^- and SO_4^{2-} were almost unaffected. This result indicates that the active site in Mg-N-C is the metal site Mg, instead of nitrogen or carbon.

1.3 Characterization of post-doped Mg in N-C host and ORR performance of the catalysts

It shows obvious catalytic activity enhancement in both alkaline and acidic environments when adding Mg and N sources (N-C-3Mg) (Supplementary Figure 32b and d) and the performance are even comparable to pre-doped Mg-N-C catalyst in last part. The control group with same mole ratio addition of Al and N source (N-C-3Al) at same condition is also synthesized and it shows much poor activity no more change than that with only N source addition (N-C-no metal). It proves the Mg-Nx moiety formed by Mg doping is active rather than that by Al doping. And more, N doping is also a little helpful (see a slight activity increase in N-C-no metal than the original N-C (N-C-pristine)) which reveals they are not the main activity contributor. To validate the activity attribution of Mg addition, different Mg source contents but same N source contents were added in N-C host. The catalyst with higher content of Mg source addition (N-C-3Mg) shows better performance than the lower one with a third of Mg addition (N-C-1Mg), and they are both much better than the catalyst with only N sources but no Mg addition (N-C-0Mg) (Supplementary Figure 32c and e), which reveals that there are more Mg-Nx moieties by increase Mg doping in N-C-3Mg and improves the activity. Moreover, even synthesized with only Mg source but no N addition in N-C host (N-C-3Mg-no N source), Mg doping can promote the activity than N-C-pristine, this is mainly contributed from the unsaturated N sites in the N-C host which give the binding sites for Mg and form the Mg-Nx moieties.

To prove the existence of Mg-Nx moieties, The XRD and FTIR results (Supplementary Figure 31), and HRTEM images (Supplementary Figure 32) proves that no metal atoms or metal oxide exists in the graphitized carbon catalysts. Furthermore, EDS images in Supplementary Figure 35 indicate that Mg is uniformly distributed in samples. The XPS results in Supplementary Figure 36a reveals that with the increment of Mg content, the intensity of Mg (2+) becomes stronger, which leads to the increment in $E_{1/2}$ under both the alkaline and acidic conditions. The activity in basic solution (Supplementary Figure 36b) and acidic solution (Supplementary Figure

36c) is directly correlated with Mg contents (red polyline) but N contents or N doping forms. Note that there is no Fe contamination in the catalysts are observed as shown in Supplementary Table 10. Thus, all the results above prove that there are Mg-Nx moieties existing in the Mg-N-C catalysts, which works as the active site in ORR tests and promotes the catalytic performance of the catalysts.

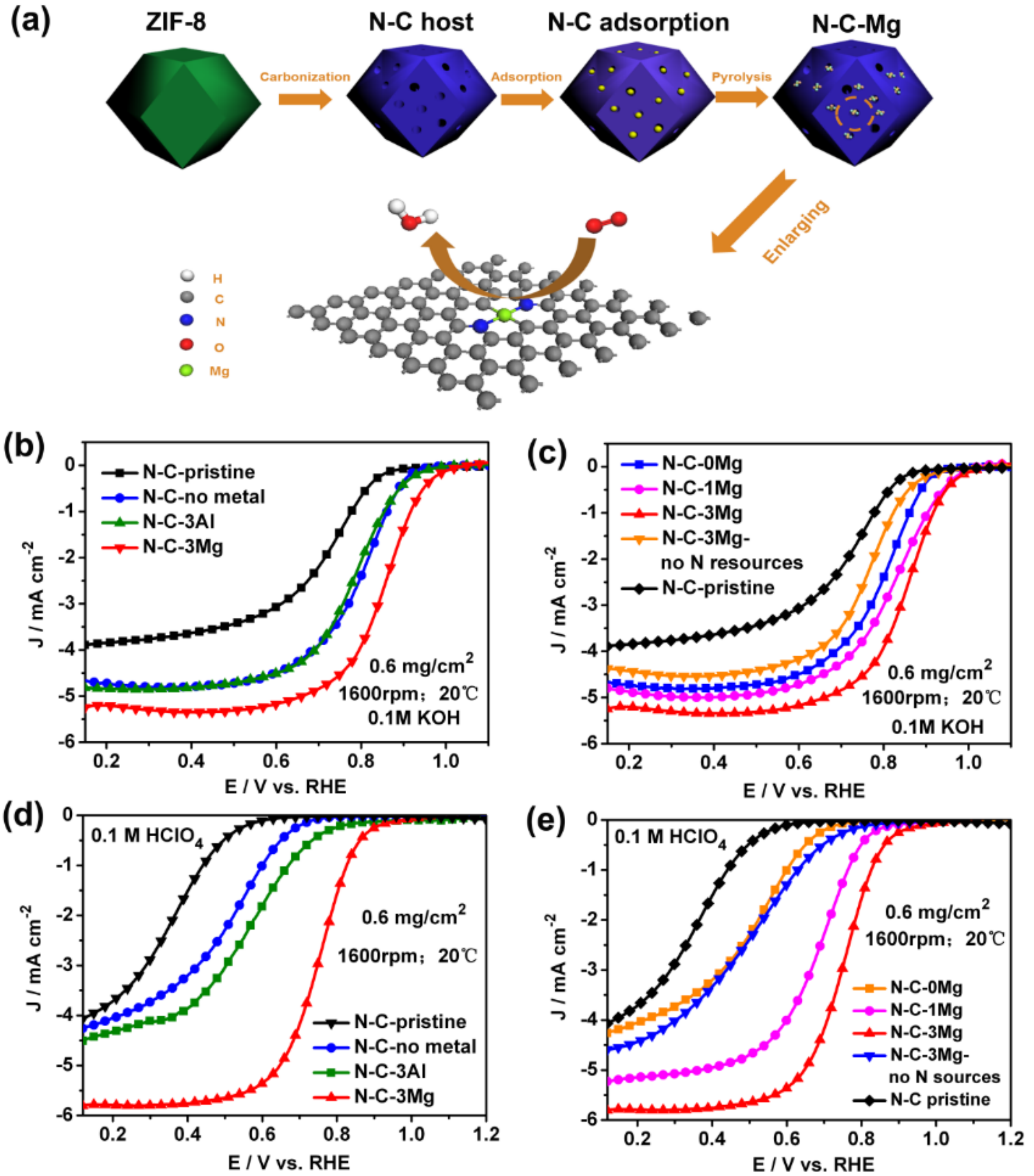

Supplementary Figure 32. The synthesis diagrams of post-doped Mg cofactor in N-C host and the relationship between ORR performance and Mg doping contents. The normalized LSV curves are recorded at a sweep rate of 10 mV s^{-1}. (a) The synthesis process diagrams, ZIF-8 is transferred into nitrogen-doped porous carbon (N-C host) after carbonization and etching, then metal and N sources are adsorbed in N-C host and

N-C-X (X represents the metal doping) is obtained after further pyrolysis. (b) The ORR performance from effect of metal-nitrogen sources addition in alkaline condition and (d) in acidic condition. (c) The ORR performance from effect of different Mg source contents addition in alkaline condition and (e) in acidic condition.

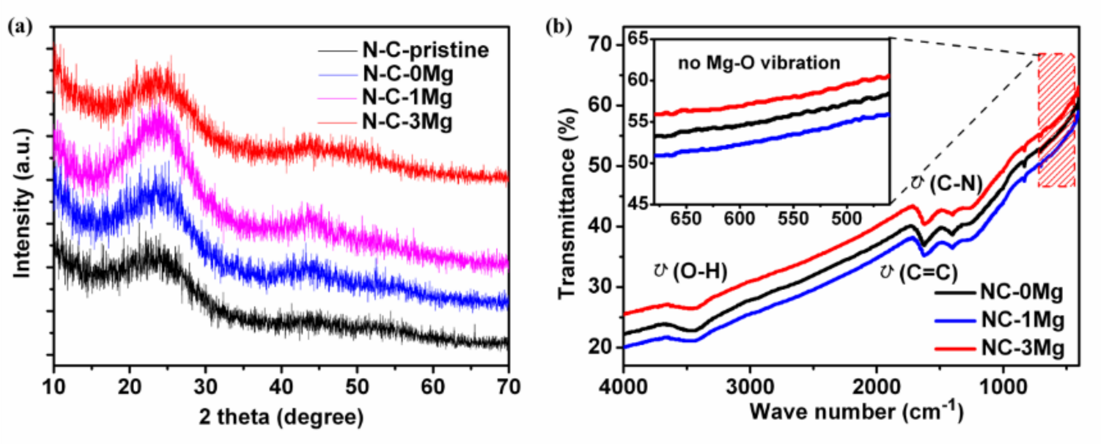

Supplementary Figure 33. The XRD patterns and FTIR spectra for the post-doped catalysts. (a) Carbon diffraction peaks appear in all four catalysts and become stronger when the Mg content increases. No metal or oxides in the final products. (b) FTIR spectra shows that no Mg-O stretching vibrations at ~500 cm^{-1} are observed, indicating that metal oxides are removed in the catalysts.

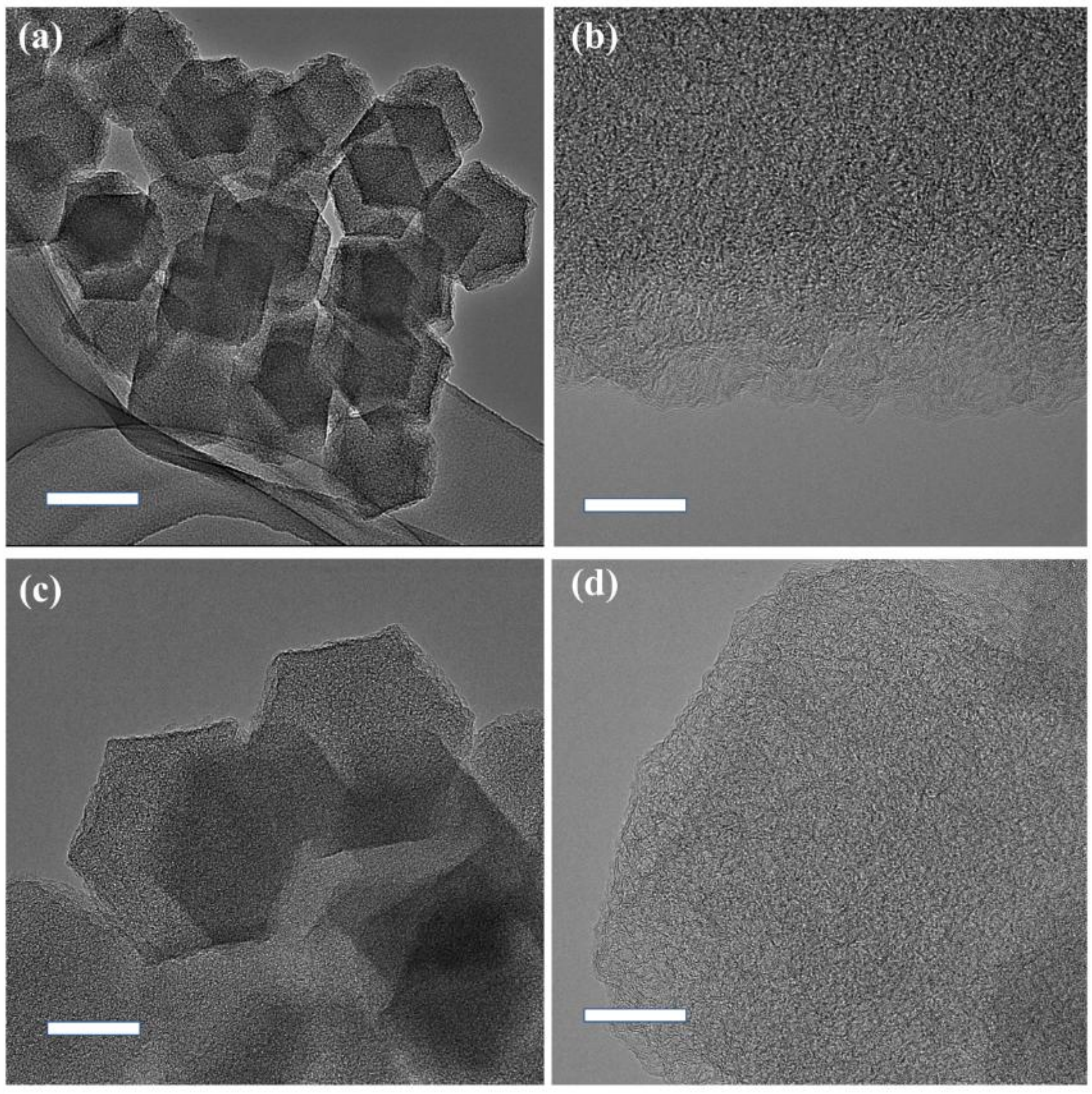

Supplementary Figure 34. The HRTEM images for post-doped graphene-based Mg-N cofactors. (a-b) the images for N-C-1Mg at different scales (a: 100 nm; b: 10 nm). (c-d) the images for N-C-3Mg at different scales (c: 50 nm; d: 10 nm). There are no metal or metal oxides are observed in the products, indicating the formation of Mg doping.

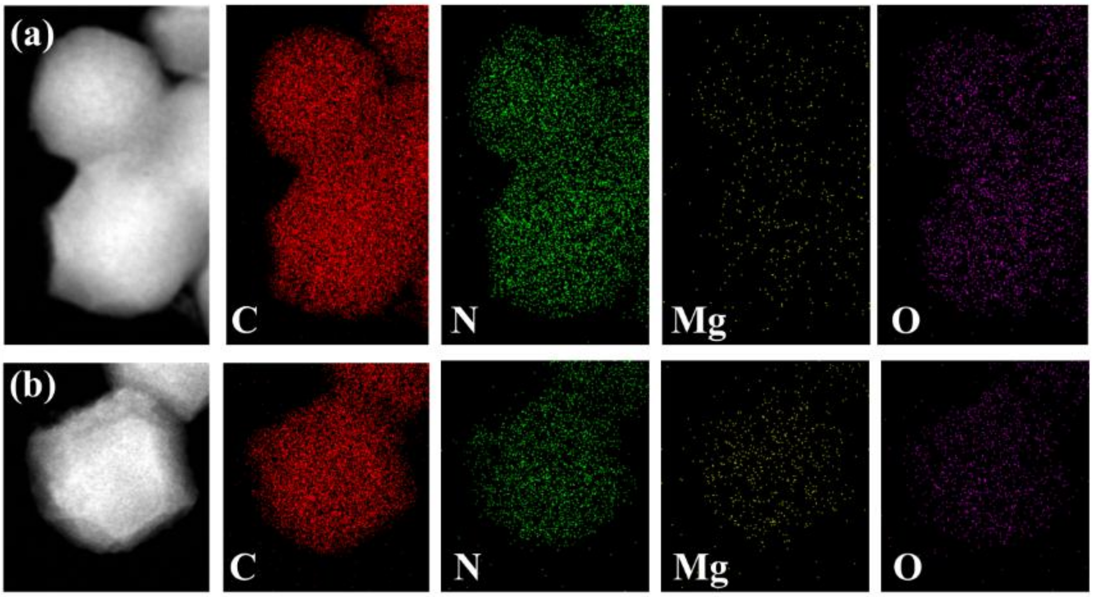

Supplementary Figure 35. EDS maps shows the uniform distribution of Mg elements in the catalysts. (a) Elements distribution on N-C-1Mg. (b) Elements distribution on N-C-3Mg. Stronger Mg signal is observed in single particle of N-C-3Mg that in N-C-1Mg, indicating larger Mg content in the catalysts.

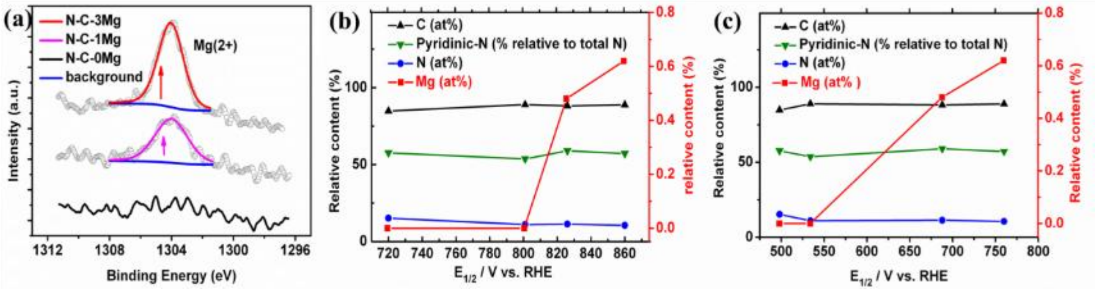

Supplementary Figure 36. The relationship between different Mg doping contents in catalysts and the ORR performance under both alkaline and acidic condition, data from XPS results. (a) With the content of Mg increases, the Mg 1s signal becomes stronger. (b) The relation of half-wave potential ($E_{1/2}$) of catalysts and elements contents in basic solution. (c) The relation of half-wave potential ($E_{1/2}$) of catalysts and elements contents in acidic solution.

The ORR performance is directly correlated with Mg contents as shown in red polyline while no more relation with C, N and Pyridinic-N doping forms. Here, although N doping slightly improves the activity (as shown in Supplementary Figure 32, the N-C-no metal is better than N-C-pristine) but contents in N-C-pristine is higher than post-doped catalysts after thermal treatment, so N doping content shows no direct

correlation with ORR activity or it is not the main active sites for ORR process in catalysts.

1.4 **Reaction pathway details and other spatial configurations similar to MN2C**

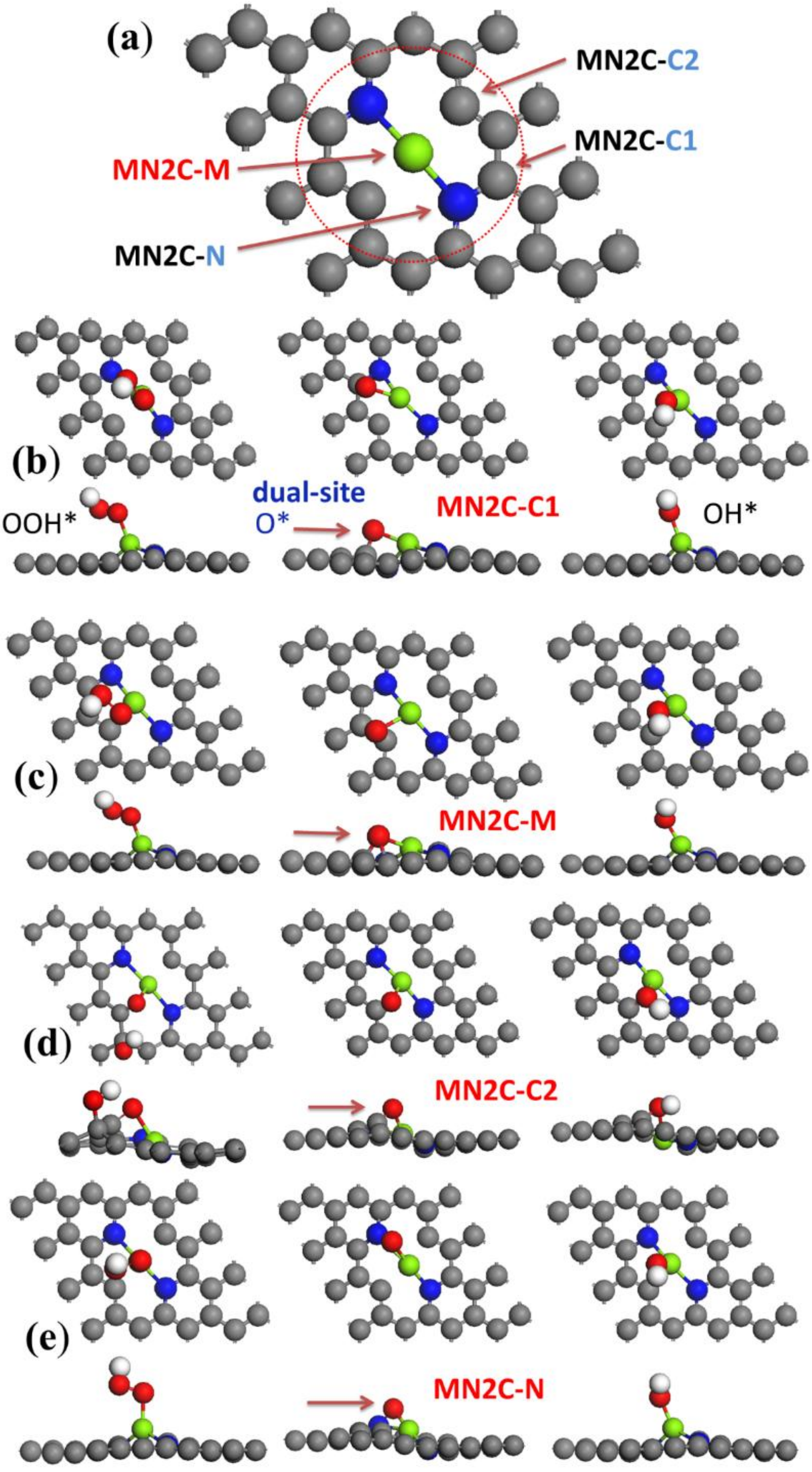

(a)
MN2C-C2
MN2C-C1
MN2C-M
MN2C-N
(b)
dual-site
OOH*
O*
MN2C-C1
OH*
(c)
MN2C-M
(d)
MN2C-C2
(e)
MN2C-N

Supplementary Figure 37. Active sites identification for MN2C. (a) Possible active sites in MN2C catalyst for the ORR. And the atomistic structures of OOH* (left panel), O*(middle panel) and OH* (right panel) when (b) C1, (c) Mg, (d) C2 and (e) N atoms are set as the active site, respectively. The O* is adsorbed at duel sites by both Mg and surrounding atoms as the red arrows pointed out. It further reveals that Mg doping not only moderates the adsorption strength of oxygen species at Mg site, but also change the adsorption strength at surrounding carbon atoms.

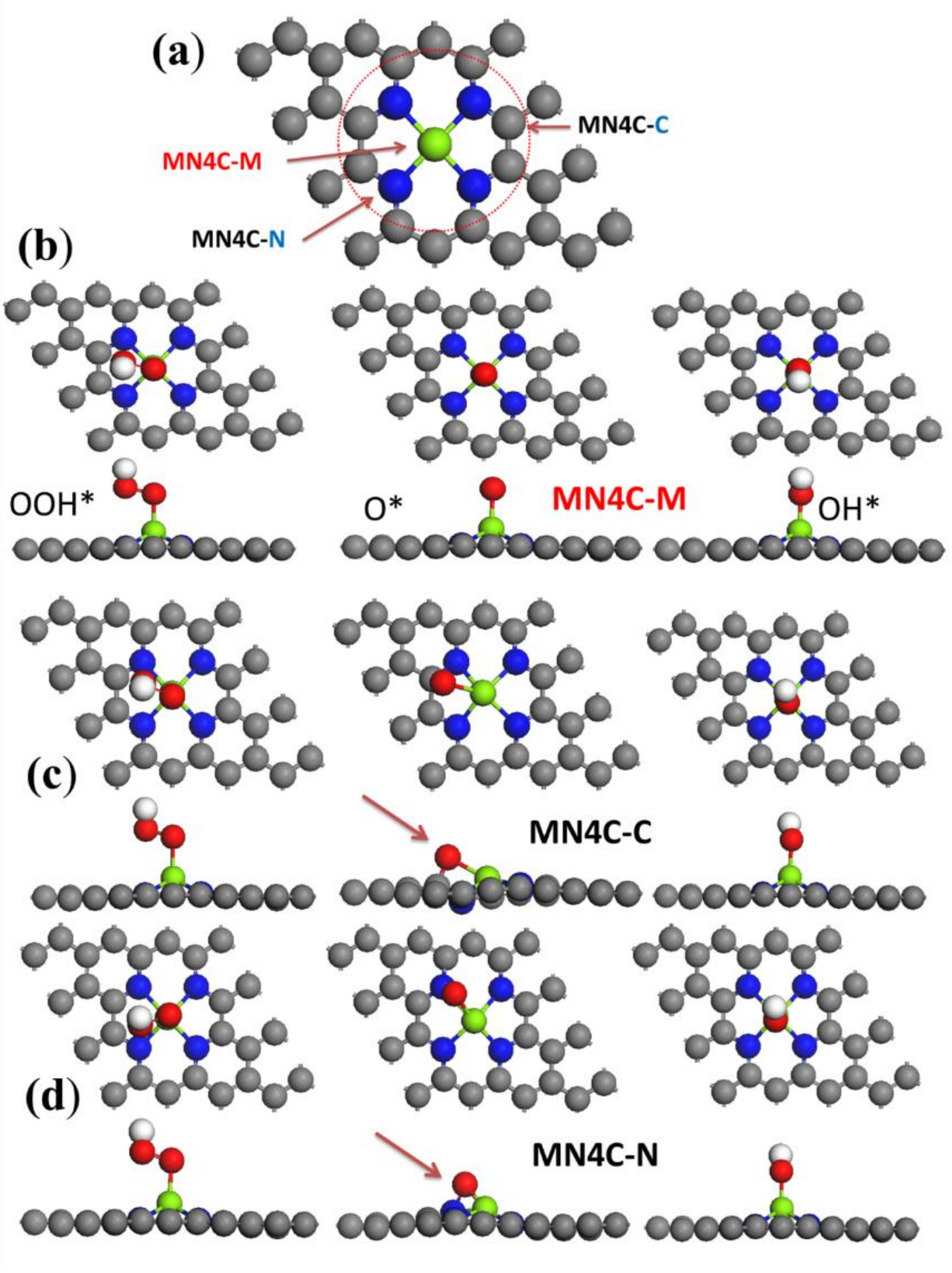

Supplementary Figure 38. Active sites identification for MN4C**.** (a) Possible active sites in MN4C catalyst for the ORR. And the atomistic structures of OOH* (left panel), O*(middle panel) and OH* (right panel) when (b) Mg, (c) C and (d) N atoms are set as the active site, respectively. The O* is adsorbed at duel sites by both Mg and surrounding atoms as the red arrows pointed out. It further reveals that Mg doping will not only moderate the adsorption strength of oxygen species at Mg site, but also change the adsorption strength at surrounding carbon atoms.

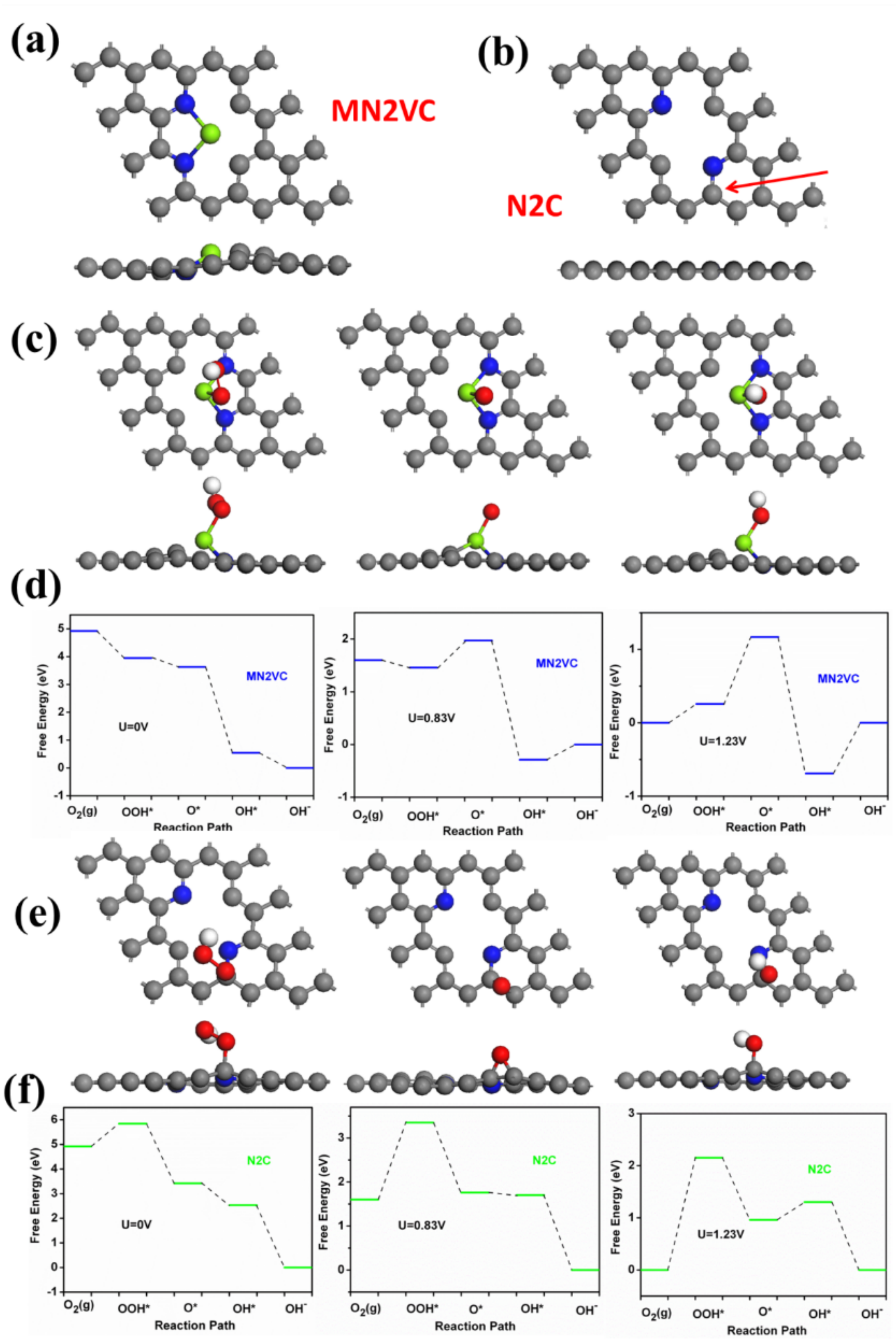

Supplementary Figure 39. Possible atomistic configuration for MN2C. (b) Atomistic structure for N2C when metallic atom is in absence. (c) Atomistic structures of initial state (left panel, state OOH*), transition state (middle panel, state O*) and final state

(right panel, state OH*) for the reactive pathway of MN2VC and (e) for N2C. (d) ORR Free energy diagram for MN2VC and (f) for N2C.

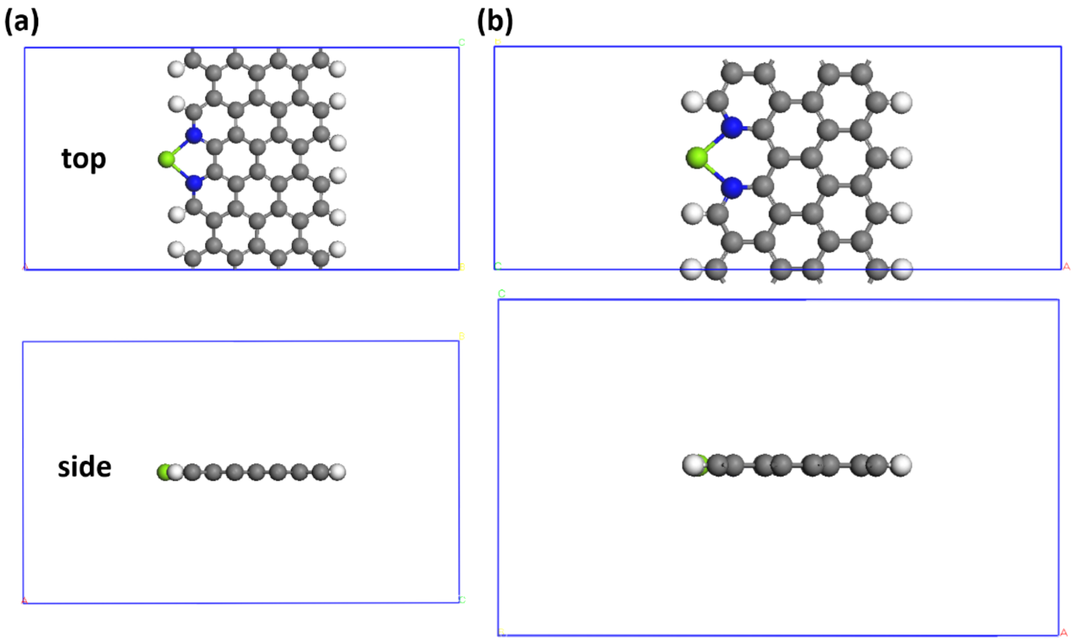

Supplementary Figure 40. Configurations of edge-bonded Mg cofactors. (a) The top and side views of armchair edge-bonded MgN2C cofactor. (b) The top and side views of zigzag edge-bonded MgN2C cofactor.

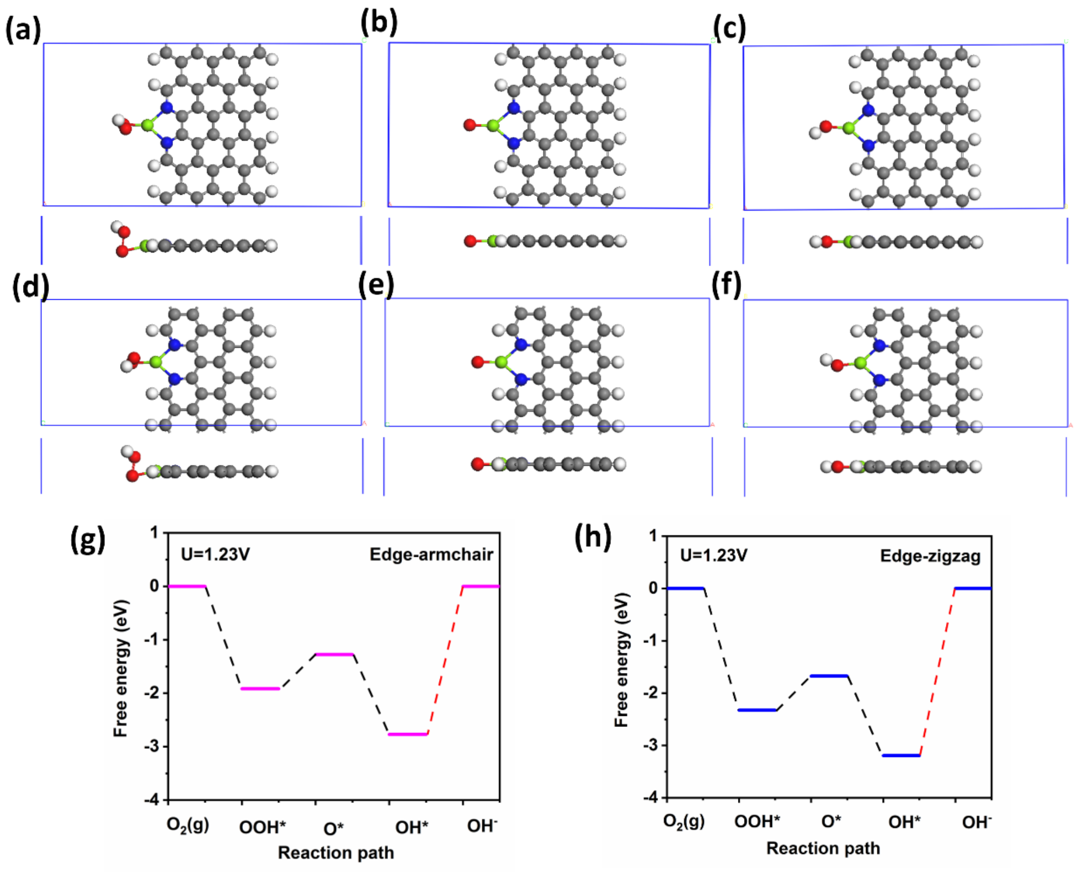

Supplementary Figure 41. The configurations of intermediates on edge-bonded Mg cofactors and corresponding free energy diagrams. (a-c) The top and side views of OOH*, O*, OH* at armchair edge-bonded MgN2C cofactor. (d-f) The top and side views of OOH*, O*, OH* at zigzag edge-bonded MgN2C cofactor. (g and h) The free energy diagrams of armchair edge-bonded MgN2C and zigzag edge bonded MgN2C.

From the result of XANES and other characterizations, the Mg center coordinated with two nitrogen atoms is the most likely active cofactor combined with DFT results, so we also calculate the other possible configuration of MN2VC, but it shows poor activity than MN2C, so MN2C is the most possible active structure for ORR. To further confirm the Mg contribution, the structure with Mg absence is also calculated, as shown in Fig. f, it shows much poor activity with so large energy barriers at oxygen activation step (O_2 transforms to OOH), thus lower onset potential (Table S4). These results confirm the importance of Mg center and the catalytic role in Mg cofactors for ORR.

To evaluate the activity contribution of the edge-bonded MgN2C cofactors, we also calculated the free energy on the armchair edge and zigzag edge Mg cofactors as shown in Supplementary Figure 40. The intermediates adsorbed on the two typical edge

cofactors are shown in Supplementary Figure 41, and the adsorption strength of OH* at the two cofactors are too strong to catalyze the ORR.

2. Supplementary Tables

Supplementary Table 1. Reaction free energies of elementary steps and predicted adsorption energies of various ORR species for reference at zero potential.

Step energy barrier (eV)	ΔG_1	ΔG_2	ΔG_3	ΔG_4
Pt	-0.84	-1.60	-1.72	-0.77
Ideal model	-1.23	-1.23	-1.23	-1.23

Adsorbates energy (eV)	ΔG_{O*}	ΔG_{OH*}	ΔG_{OOH*}
Pt	-2.34	-0.84	-3.9
Ideal model	-2.46	-1.23	-3.69

Supplementary Table 2. Reaction free energies of elementary steps for MNxC, ANxC and CNxC at zero potential, where the metallic center is set as the active sites and the reaction free energies on possible active sites of the MN2C and MN4C catalysts.

Step energy barrier for Mg center (eV)	ΔG_1	ΔG_2	ΔG_3	ΔG_4
MN4C	-1.09	-0.41	-2.97	-0.45
MN2C	-0.84	-1.60	-1.72	-0.77
MN3C	-5.94	-0.48	-2.87	4.37
MN1C	-6.85	-0.46	-2.90	-5.28

Step energy barrier for Al center (eV)	ΔG_1	ΔG_2	ΔG_3	ΔG_4
AN4C	-2.51	-0.97	-2.69	-1.25
AN2C	-1.53	-0.97	-2.64	0.22
AN3C	-5.60	-1.83	-1.79	-4.29
AN1C	-4.24	-1.19	-2.65	-3.15

Step energy barrier for Ca center (eV)	ΔG_1	ΔG_2	ΔG_3	ΔG_4
CN4C	-1.49	-0.32	-2.95	-0.16
CN2C	-1.36	-0.32	-2.88	-0.37
CN3C	-11.55	-0.25	-3.00	9.89
CN1C	-11.52	0.68	-3.92	9.85

Step energy barrier (eV)	ΔG_1	ΔG_2	ΔG_3	ΔG_4
MN2C	-0.84	-1.60	-1.72	-0.77
MN2VC	-0.97	-0.32	-3.09	-0.54
N2C	0.92	-2.42	-0.89	-2.53

Step energy barrier (eV)	ΔG_1	ΔG_2	ΔG_3	ΔG_4
MN2C-M	-0.84	-1.60	-1.72	-0.77
MN2C-C1	-0.82	-0.96	-2.31	-0.84
MN2C-C2	-3.25	-2.10	0.45	-0.02

MN2C-N	-0.70	-1.37	-2.00	-0.85

Step energy barrier (eV)	ΔG_1	ΔG_2	ΔG_3	ΔG_4
MN4C-M	-1.09	-0.41	-2.97	-0.45
MN4C-C	-1.09	-1.26	-2.12	-0.45
MN4C-N	-1.09	-0.84	-2.54	-0.45

Supplementary Table 3. Predicted adsorption energies of various ORR species for MNxC, ANxC and CNxC at zero potential, where the metallic center is set as the active sites and the predicted adsorption energies on possible active sites of the MN2C and MN4C catalysts

Adsorbates energy for Mg center (eV)	ΔG_{O*}	ΔG_{OH*}	ΔG_{OOH*}
MN4C	3.42	0.45	3.83
MN2C	2.48	0.77	4.08
MN3C	-1.50	-4.37	-1.02
MN1C	-2.39	-5.28	-1.93

Adsorbates energy for Al center (eV)	ΔG_{O*}	ΔG_{OH*}	ΔG_{OOH*}
AN4C	1.44	-1.25	2.41
AN2C	2.42	-0.22	3.39
AN3C	-2.51	-4.29	-0.68
AN1C	-0.51	-3.15	0.68

Adsorbates energy for Ca center (eV)	ΔG_{O*}	ΔG_{OH*}	ΔG_{OOH*}

CN4C	3.12	0.16	3.43
CN2C	3.24	0.37	3.56
CN3C	-6.88	-9.89	-6.63
CN1C	-5.93	-9.85	-6.60

Adsorbates energy (eV)	ΔG_{O*}	ΔG_{OH*}	ΔG_{OOH*}
MN2C	2.48	0.77	4.08
MN2VC	3.63	0.54	3.95
N2C	3.42	2.53	5.84

Adsorbates energy (eV)	ΔG_{O*}	ΔG_{OH*}	ΔG_{OOH*}
MN2C-M	2.48	0.77	4.08
MN2C-C1	3.15	0.84	4.10
MN2C-C2	-0.42	0.02	1.67
MN2C-N	2.85	0.85	4.22

Adsorbates energy (eV)	ΔG_{O*}	ΔG_{OH*}	ΔG_{OOH*}
MN4C-M	3.42	0.45	3.83
MN4C-C	2.57	0.45	3.83
MN4C-N	2.30	0.45	3.83

Supplementary Table 4. Calculated onset potential values for different models.

models	$U_{\mathrm{RHE}}^{\mathrm{onset}}$

	(V vs. RHE)
MN1C	-5.28
MN2C	**0.77**
MN3C	-4.37
MN4C	**0.41**
AN1C	-3.15
AN2C	-0.22
AN3C	-4.29
AN4C	-1.25
CN1C	-9.85
CN2C	**0.32**
CN3C	-9.89
CN4C	0.16

models	$U_{\mathrm{RHE}}^{\mathrm{onset}}$ (V)
Pt	0.84
Ideal model	1.23

models	$U_{\mathrm{RHE}}^{\mathrm{onset}}$ (V)
MN2C	**0.77**
MN2VC	0.32
N2C	-0.92

models	$U_{\mathrm{RHE}}^{\mathrm{onset}}$ (V)
MN2C-M	0.77
MN2C-C1	**0.82**

MN2C-C2	-0.45
MN2C-N	0.70

models	$U_{\mathrm{RHE}}^{\mathrm{onset}}$ (V)
MN4C-M	0.41
MN4C-C	**0.45**
MN4C-N	0.45

Supplementary Table 5. Theoretical onset potential for the TM-N4-C models (data obtained from the literature reported [25]).

Models	ΔG_{OH*} (eV)	$U_{\mathrm{RHE}}^{\mathrm{onset}}$ (V)
Fe	0.81	**0.81**
Co	1.14	**0.73**
Ni	1.56	0.63
Cu	1.71	0.66
Zn	2.06	-0.10
Cr	0.03	0.03
Mn	0.15	0.25
Cd	1.89	-0.07
Ru	1.47	0.34
Rh	1.34	0.46
Pd	2.54	-0.41
Ag	1.60	0.48

Supplementary Table 6. Adsorbate strength and the p-electrons feature.

(A) The outer shell p-electrons integrated for all models at metal sites

models	Spin-up (state)	Spin-down (state)
MN2C	0.098	0.098
MN4C	0.116	0.116
MN3C	0.175	0.175
MN1C	1.37	1.48

(B) The Adsorption free energies of OH as a function of the ε_p position of metal centers for Mg, Al and Ca cofactors.

Models	P band center position (eV)	ΔG_{OH*} (eV)
MN2C	5.99	0.77
MN4C	4.65	0.45
MN3C	2.25	-4.37
AN2C	5.75	-0.22
AN4C	2.50	-1.25
AN3C	0.51	-4.29
CN2C	-4.87	0.40
CN4C	-4.33	0.16
CN3C	-12.16	-9.89

(C) The Bader charge analysis and electrons donation from OH radical to MNxC.

Charge value (e^-)	MN2C	MN4C	MN3C
Mg	0.3741	0.3589	0.3626
**O**	**7.3715**	**7.4055**	**7.4414**
H	0.4037	0.4097	0.3933

OH	7.7752	7.8152	7.8347
**electrons donation**	**0.2248**	**0.1848**	**0.1653**

(D) The Adsorption free energies of OH as a function of highest O occupied state of hydroxyl after interaction with Mg, Al and Ca cofactors.

Models	O 2p highest occupied level after interaction (eV)	ΔG_{OH*} (eV)
MN2C-OH	-0.16	0.77
MN4C-OH	-0.45	0.45
MN3C-OH	-0.8	-4.37
AN2C-OH	-1	-0.22
AN4C-OH	-2	-1.25
AN3C-OH	-4	-4.29
CN2C-OH	-0.15	0.37
CN4C-OH	-0.5	0.16
CN3C-OH	-4.2	-9.89

Supplementary Table 7. The crystal data for Mg-HMT.

Lattice parameters:

a	b	c	alpha	beta	gamma
**9.50800**	9.42800	9.30900	78.8600	105.0800	120.1300

Unit-cell volume = 694.910380 Å^3

Supplementary Table 8. Elements contents of Mg-N-C from ICP-AES analysis.

Elements (wt%)	Mg	Fe	Co	Ni
Mg-N-C	1.30	0.018	0.001	0.001

Supplementary Table 9. The ORR activity comparison between Mg-N-C and other catalysts reported in 0.1 M KOH electrode and acidic solution.

The alkaline ORR activity comparison between this work and reported materials in recent years.

**Reported time**	**Co-based electrocatalysts**	**Half-wave potential (V vs. RHE)**	**References**
2015.10	Co-N-C-0.8 NPHs	0.871	[26]
2016.1	ZIF-67 derived carbon	0.87	[27]
2016.6	CoSAs/N-C(900)	0.88	[28]
2017	NC@Co-NGC DSNC	0.82	[29]
2017.2	Co-C3N4/CNT	0.85	[30]
2018.7	Co3O4/HNCP-40	0.845	[31]
2018.11	N-C-CoO_x	0.84	[32]
2019.9	Co-N-C	0.91	[33]
**2019.9**	**Mg-N-C**	**0.91**	**This work**

**Reported time**	**Fe-based electrocatalysts**	**Half-wave potential (V vs. RHE)**	**References**

2017.4	Fe-N-C	0.90	[34]
2018.10	CNT/PC	0.88	[35]
2019	CAN-Pc(Fe/Co)	0.84	[36]
2019.6	Co–Fe alloy	0.89	[37]
2019.9	Fe–NCNWs	0.91	[38]
**2019.9**	**Mg-N-C**	**0.91**	**This work**

**Reported time**	**Mn or Cu-based electrocatalysts**	**Half-wave potential (V vs. RHE)**	**References**
2016.9	Cu–N@C	0.80	[39]
2018.6	Mn-N-O	0.86	[40]
2019.5	Mn@NG	0.82	[41]
2019.8	Cu-N-C-ICHP	0.85	[42]
2019.8	Cu ISAS/NC	0.92	[43]
2019.9	Cu/G	0.85	[44]
**2019.9**	**Mg-N-C**	**0.91**	**This work**

**Reported time**	**Carbon-based electrocatalysts**	**Half-wave potential (V vs. RHE)**	**References**
2016,12	N,S co-doped carbon	0.87	[45]
2017.1	1100-CNS	0.85	[46]

2017.7	NHPC-900-1000	0.84	47
2018.2	N-Doped Carbons	0.69	48
2018.10	NFLGDY-900c	0.87	49
2018.10	N-HC@G-900	0.85	50
2019.9	SNBCs	0.85	51
**2019.9**	**Mg-N-C**	**0.91**	**This work**

The corresponding chart for alkaline ORR activity comparison.

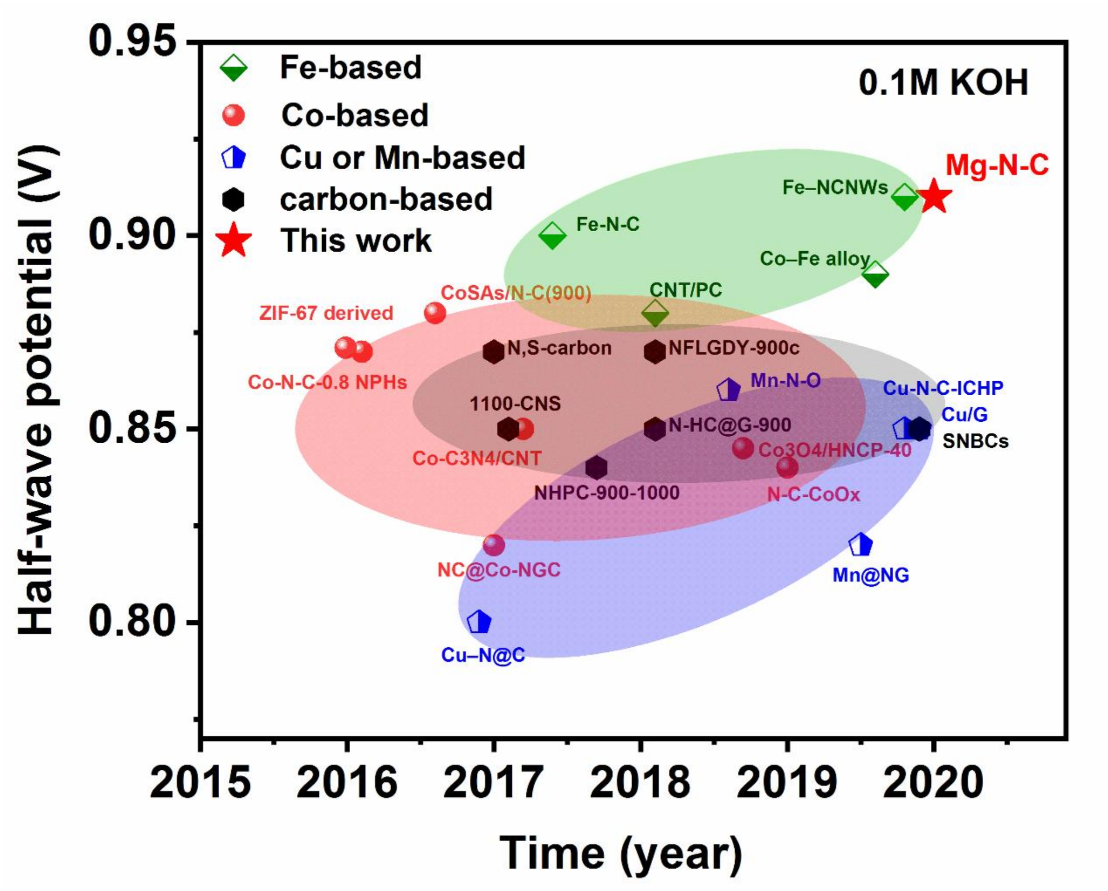

The acidic ORR activity comparison between this work and reported materials in recent years.

**Reported time**	**Fe-based electrocatalysts**	**Half-wave potential (V vs. RHE)**	**References**
2015.5	Fe-N-C nanofiber	0.62	[52]
2015.6	Fe-N-C	0.84	[53]
2016.7	Fe-N-C	0.82	[54]
2017.8	(CM+PANI) Fe-C	0.80	[55]
2017.11	Fe SAs/N-C	0.75	[56]
2018.10	CNT/PC	0.79	[35]
2018.12	PF-2	0.771	[57]
2019.6	Fe-N-C	0.88	[58]
2019.9	Fe–NCNWs	0.82	[38]
**2019.9**	**Mg-N-C**	**0.79**	**This work**

**Reported time**	**Co, Mn or Cr-based electrocatalysts**	**Half-wave potential (V vs. RHE)**	**References**
2015.10	Co-N-C-0.8 NPHs	0.761	[26]
2017.11	Co SAs/N-C	0.747	[56]
2018.10	Mn-N-C-second	0.80	[59]
2018.10	Mn-N-C	0.78	[60]
2019.7	Cr-N-C	0.774	[61]
**2019.9**	**Mg-N-C**	**0.79**	**This work**

Reported time	Carbon-based electrocatalysts	Half-wave potential (V vs. RHE)	References
2015.10	N, P-doped porous carbon	0.62	[62]
2016.4	N, P-doped CGHNs	0.68	[63]
2017.1	1100-CNS	0.73	[46]
2018.10	N-HC@G-900	0.65	[50]
**2019.9**	**Mg-N-C**	**0.79**	**This work**

The corresponding chart for alkaline ORR activity comparison.

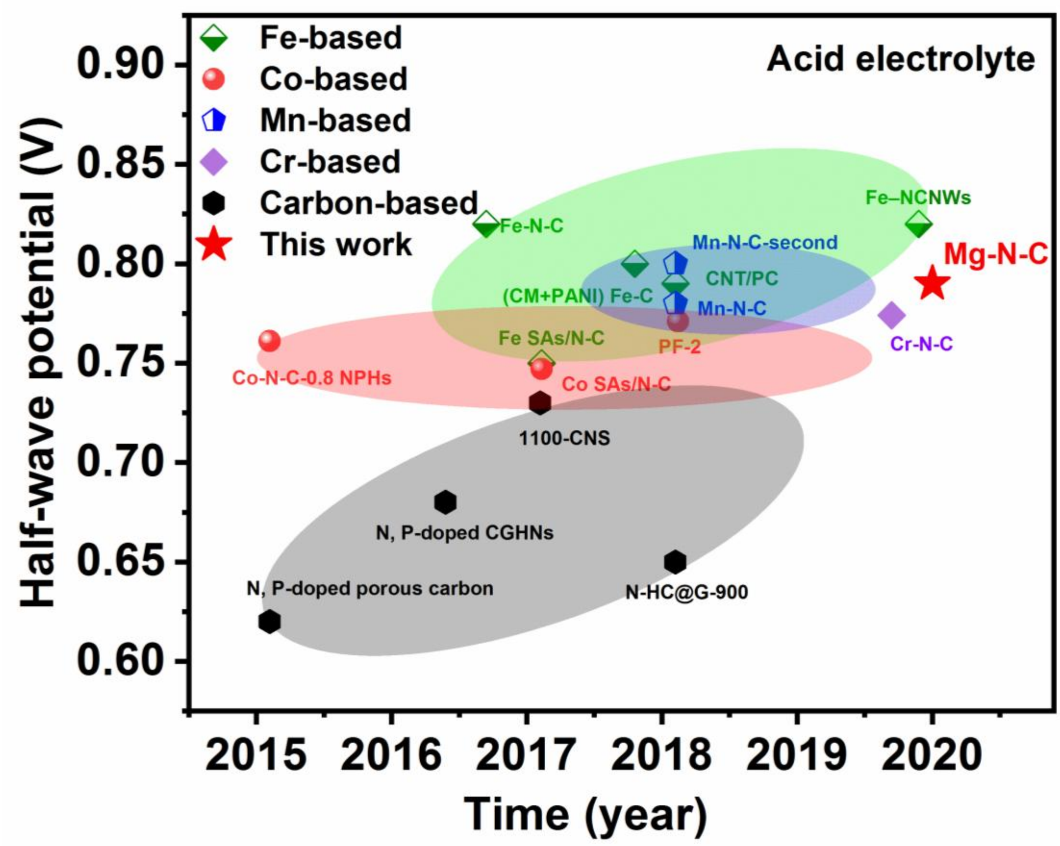

Supplementary Table 10. Elements contents of post-doped N-C-X from XPS analysis.

Elements (wt%)	C	N	Mg	Fe
N-C-pristine	84.78	15.22	0	0
N-C-0Mg	88.99	11.01	0	0
N-C-1Mg	88.18	11.35	0.48	0
N-C-3Mg	88.85	11.54	0.62	0

Supplementary Table 11. The entropy and zero-point energy corrections values used for the calculation for the structure similarity and the values for the different metal atoms are rather close.

species	T × S (eV) (298K)	ZPE (eV)
H2	0.40	0.27
H2O	0.67	0.56
O*	0	0.05
OOH*	0	0.35
OH*	0.01	0.31

3. Supplementary References

1. Zheng, F., Yang, Y. & Chen, Q. High lithium anodic performance of highly nitrogen-doped porous carbon prepared from a metal-organic framework. *Nature Communications* **5**, 5261 (2014).
2. Ferrari, A. C. & Basko, D. M. Raman spectroscopy as a versatile tool for studying the properties of graphene. *Nature nanotechnology* **8**, 235 (2013).

3. Ingason, A. S., Eriksson, A. K., Lewin, E., Jensen, J. & Olafsson, S. Growth and structural properties of Mg:C thin films prepared by magnetron sputtering. *Thin Solid Films* **518**, 4225–4230 (2010).
4. Ramachandran, M. & Reddy, R. G. Direct reduction of magnesium oxide to magnesium using thermal plasma technology. *Mining, Metallurgy & Exploration* **32**, 30–37 (2015).
5. Petnikota, S. *et al.* Electrochemistry-related aspects of safety of graphene-based non-aqueous electrochemical supercapacitors: a case study with MgO-decorated few-layer graphene as an electrode material. *New J. Chem.* **43**, 9793–9801 (2019).
6. Yang, Y., Zheng, F., Xia, G., Lun, Z. & Chen, Q. Experimental and theoretical investigations of nitro-group doped porous carbon as a high performance lithium-ion battery anode. *Journal of Materials Chemistry A* **3**, 18657–18666 (2015).
7. Huang, H.-H., Shih, W.-C. & Lai, C.-H. Nonpolar resistive switching in the Pt/MgO/Pt nonvolatile memory device. *Applied Physics Letters* **96**, 193505 (2010).
8. Le Febvrier, A., Jensen, J. & Eklund, P. Wet-cleaning of MgO (001): Modification of surface chemistry and effects on thin film growth investigated by x-ray photoelectron spectroscopy and time-of-flight secondary ion mass spectroscopy. *Journal of Vacuum Science & Technology A: Vacuum, Surfaces, and Films* **35**, 021407 (2017).

9. Zhang, H. *et al.* Single atomic iron catalysts for oxygen reduction in acidic media: particle size control and thermal activation. *Journal of the American Chemical Society* **139**, 14143–14149 (2017).

10. Jansson, S., Brabie, V. & Jönsson, P. Corrosion Mechanism of Commercial MgO–C Refractories in Contact with Different Gas Atmospheres. *ISIJ Int.* **48**, 760–767 (2008).

11. Fruehan, R. J. & Martonik, L. J. The Rate of reduction of MgO by carbon. *MTB* **7**, 537–542 (1976).

12. Li, J. *et al.* Thermally Driven Structure and Performance Evolution of Atomically Dispersed FeN4 Sites for Oxygen Reduction. *Angewandte Chemie International Edition* **n/a**, (2019).

13. Liu, Q., Liu, X., Zheng, L. & Shui, J. The Solid-Phase Synthesis of an Fe-N-C Electrocatalyst for High-Power Proton-Exchange Membrane Fuel Cells. *Angew. Chem. Int. Ed.* **57**, 1204–1208 (2018).

14. Yang, W. *et al.* Facile Synthesis of Fe/N/S-Doped Carbon Tubes as High-Performance Cathode and Anode for Microbial Fuel Cells. *ChemCatChem* **n/a**, (2019).

15. Li, Z. Q., Lu, C. J., Xia, Z. P., Zhou, Y. & Luo, Z. X-ray diffraction patterns of graphite and turbostratic carbon. *Carbon* **45**, 1686–1695 (2007).

16. Choudhury, B. & Choudhury, A. Microstructural, optical and magnetic properties study of nanocrystalline MgO. *Materials Research Express* **1**, 025026 (2014).

17. Wu, G., More, K. L., Johnston, C. M. & Zelenay, P. High-Performance Electrocatalysts for Oxygen Reduction Derived from Polyaniline, Iron, and Cobalt. *Science* **332**, 443–447 (2011).
18. Li, Z. *et al.* Ionic Liquids as Precursors for Efficient Mesoporous Iron‐Nitrogen‐Doped Oxygen Reduction Electrocatalysts. *Angewandte Chemie International Edition* **54**, 1494–1498 (2015).
19. Wang, Q. *et al.* Phenylenediamine-based FeN x/C catalyst with high activity for oxygen reduction in acid medium and its active-site probing. *Journal of the American chemical Society* **136**, 10882–10885 (2014).
20. Oh, I., Biggin, M. E. & Gewirth, A. A. Poisoning the active site of electrochemical reduction of dioxygen on metal monolayer modified electrode surfaces. *Langmuir* **16**, 1397–1406 (2000).
21. Cheng, Y., Zhang, J. & Jiang, S. P. Are metal-free pristine carbon nanotubes electrocatalytically active? *Chemical Communications* **51**, 13764–13767 (2015).
22. Thorum, M. S., Hankett, J. M. & Gewirth, A. A. Poisoning the oxygen reduction reaction on carbon-supported Fe and Cu electrocatalysts: evidence for metal-centered activity. *The Journal of Physical Chemistry Letters* **2**, 295–298 (2011).
23. Guo, D. *et al.* Active sites of nitrogen-doped carbon materials for oxygen reduction reaction clarified using model catalysts. *Science* **351**, 361–365 (2016).
24. Jia, Y. *et al.* Identification of active sites for acidic oxygen reduction on carbon catalysts with and without nitrogen doping. *Nature Catalysis* **2**, 688–695 (2019).

25. Xu, H., Cheng, D., Cao, D. & Zeng, X. C. A universal principle for a rational design of single-atom electrocatalysts. *Nature Catalysis* **1**, 339–348 (2018).

26. You, B. *et al.* Bimetal–organic framework self-adjusted synthesis of support-free nonprecious electrocatalysts for efficient oxygen reduction. *Acs Catalysis* **5**, 7068–7076 (2015).

27. Xia, B. Y. *et al.* A metal–organic framework-derived bifunctional oxygen electrocatalyst. *Nat Energy* **1**, 1–8 (2016).

28. Yin, P. *et al.* Single Cobalt Atoms with Precise N-Coordination as Superior Oxygen Reduction Reaction Catalysts. *Angewandte Chemie International Edition* **55**, 10800–10805 (2016).

29. Liu, S. *et al.* Metal-Organic-Framework-Derived Hybrid Carbon Nanocages as a Bifunctional Electrocatalyst for Oxygen Reduction and Evolution. *Adv. Mater.* **29**, 1700874 (2017).

30. Zheng, Y. *et al.* Molecule-level g-C3N4 coordinated transition metals as a new class of electrocatalysts for oxygen electrode reactions. *Journal of the American Chemical Society* **139**, 3336–3339 (2017).

31. Ding, D. *et al.* Multi-Level Architecture Optimization of MOF-Templated Co-Based Nanoparticles Embedded in Hollow N-Doped Carbon Polyhedra for Efficient OER and ORR. *ACS Catal.* **8**, 7879–7888 (2018).

32. Peng, X. *et al.* Nitrogen‐doped Carbon–CoOx Nanohybrids: A Precious Metal Free Cathode that Exceeds 1.0 W cm−2 Peak Power and 100 h Life in Anion‐Exchange Membrane Fuel Cells. *Angewandte Chemie* **131**, 1058–1063 (2019).

33. Zhu, Y. *et al.* Engineering Local Coordination Environments of Atomically Dispersed and Heteroatom‐Coordinated Single Metal Site Electrocatalysts for Clean Energy‐Conversion. *Adv. Energy Mater.* 1902844 (2019) doi:10.1002/aenm.201902844.

34. Chen, Y. *et al.* Isolated single iron atoms anchored on N‐doped porous carbon as an efficient electrocatalyst for the oxygen reduction reaction. *Angewandte Chemie International Edition* **56**, 6937–6941 (2017).

35. Sa, Y. J. *et al.* A General Approach to Preferential Formation of Active Fe–Nx Sites in Fe–N/C Electrocatalysts for Efficient Oxygen Reduction Reaction. *J. Am. Chem. Soc.* **138**, 15046–15056 (2016).

36. Yang, S. *et al.* Two‐dimensional conjugated aromatic networks as high‐site‐density and single‐atom electrocatalysts towards oxygen reduction reaction. *Angewandte Chemie International Edition* (2019) doi:10.1002/anie.201908023.

37. Xiong, Y., Yang, Y., DiSalvo, F. J. & Abruña, H. D. Metal–Organic-Framework-Derived Co–Fe Bimetallic Oxygen Reduction Electrocatalysts for Alkaline Fuel Cells. *J. Am. Chem. Soc.* **141**, 10744–10750 (2019).

38. Li, J.-C. *et al.* Secondary-Atom-Assisted Synthesis of Single Iron Atoms Anchored on N-Doped Carbon Nanowires for Oxygen Reduction Reaction. *ACS Catal.* **9**, 5929–5934 (2019).

39. Wu, H. *et al.* Highly doped and exposed Cu(I)–N active sites within graphene towards efficient oxygen reduction for zinc–air batteries. *Energy Environ. Sci.* **9**, 3736–3745 (2016).

40. Yang, Y. *et al.* O‐, N‐Atoms‐Coordinated Mn Cofactors within a Graphene Framework as Bioinspired Oxygen Reduction Reaction Electrocatalysts. *Advanced Materials* **30**, (2018).

41. Bai, L., Duan, Z., Wen, X., Si, R. & Guan, J. Atomically dispersed manganese-based catalysts for efficient catalysis of oxygen reduction reaction. *Applied Catalysis B: Environmental* **257**, 117930 (2019).

42. Wang, T. *et al.* Cu, N‐Codoped Carbon Nanodisks with Biomimic Stomata‐Like Interconnected Hierarchical Porous Topology as Efficient Electrocatalyst for Oxygen Reduction Reaction. *Small* (2019).

43. Yang, Z. *et al.* Directly transforming copper (I) oxide bulk into isolated single-atom copper sites catalyst through gas-transport approach. *Nat Commun* **10**, 3734 (2019).

44. Han, G. *et al.* High loading single-atom Cu dispersed on graphene for efficient oxygen reduction reaction. *Nano Energy* **66**, 104088 (2019).

45. Hu, C. & Dai, L. Multifunctional Carbon-Based Metal-Free Electrocatalysts for Simultaneous Oxygen Reduction, Oxygen Evolution, and Hydrogen Evolution. *Advanced Materials* **29**, 1604942 (2017).

46. Pei, Z. *et al.* Texturing in situ: N,S-enriched hierarchically porous carbon as a highly active reversible oxygen electrocatalyst. *Energy Environ. Sci.* **10**, 742–749 (2017).

47. Wu, M. *et al.* A Facile Activation Strategy for an MOF-Derived Metal-Free Oxygen Reduction Reaction Catalyst: Direct Access to Optimized Pore Structure and Nitrogen Species. *ACS Catal.* **7**, 6082–6088 (2017).

48. Qiao, M. *et al.* Low-Cost Chitosan-Derived N-Doped Carbons Boost Electrocatalytic Activity of Multiwall Carbon Nanotubes. *Advanced Functional Materials* **28**, 1707284 (2018).

49. Zhao, Y. *et al.* Few-layer graphdiyne doped with sp-hybridized nitrogen atoms at acetylenic sites for oxygen reduction electrocatalysis. *Nature Chem* **10**, 924–931 (2018).

50. Sun, J. *et al.* Ultrathin Nitrogen-Doped Holey Carbon@Graphene Bifunctional Electrocatalyst for Oxygen Reduction and Evolution Reactions in Alkaline and Acidic Media. *Angewandte Chemie* **130**, 16749–16753 (2018).

51. Kim, M.-J. *et al.* Biomass-Derived Air Cathode Materials: Pore-Controlled S,N-Co-doped Carbon for Fuel Cells and Metal–Air Batteries. *ACS Catal.* **9**, 3389–3398 (2019).

52. Wu, Z.-Y. *et al.* Iron Carbide Nanoparticles Encapsulated in Mesoporous Fe-N-Doped Carbon Nanofibers for Efficient Electrocatalysis. *Angewandte Chemie International Edition* **54**, 8179–8183 (2015).

53. Sahraie, N. R. *et al.* Quantifying the density and utilization of active sites in non-precious metal oxygen electroreduction catalysts. *Nature Communications* **6**, 8618 (2015).

54. Wang, X. *et al.* Directly converting Fe-doped metal–organic frameworks into highly active and stable Fe-N-C catalysts for oxygen reduction in acid. *Nano Energy* **25**, 110–119 (2016).

55. Chung, H. T. *et al.* Direct atomic-level insight into the active sites of a high-performance PGM-free ORR catalyst. *Science* **357**, 479–484 (2017).

56. Wang, J. *et al.* Design of N-Coordinated Dual-Metal Sites: A Stable and Active Pt-Free Catalyst for Acidic Oxygen Reduction Reaction. *J. Am. Chem. Soc.* **139**, 17281–17284 (2017).

57. Chong, L. *et al.* Ultralow-loading platinum-cobalt fuel cell catalysts derived from imidazolate frameworks. *Science* **362**, 1276–1281 (2018).

58. Zhang, H. *et al.* High-performance fuel cell cathodes exclusively containing atomically dispersed iron active sites. *Energy Environ. Sci.* **12**, 2548–2558 (2019).

59. Li, J. *et al.* Atomically dispersed manganese catalysts for oxygen reduction in proton-exchange membrane fuel cells. *Nature Catalysis* **1**, 935–945 (2018).

60. Mn- and N- doped carbon as promising catalysts for oxygen reduction reaction: Theoretical prediction and experimental validation. *Applied Catalysis B: Environmental* **243**, 195–203 (2019).

61. Luo, E. *et al.* Single-Atom Cr−N4 Sites Designed for Durable Oxygen Reduction Catalysis in Acid Media. *Angewandte Chemie International Edition* **58**, 12469–12475 (2019).

62. Zhang, J., Zhao, Z., Xia, Z. & Dai, L. A metal-free bifunctional electrocatalyst for oxygen reduction and oxygen evolution reactions. *Nature Nanotech* **10**, 444–452 (2015).

63. Yang, J. *et al.* A Highly Efficient Metal-Free Oxygen Reduction Electrocatalyst Assembled from Carbon Nanotubes and Graphene. *Advanced Materials* **28**, 4606–4613 (2016).
